# High resolution spatiotemporal modeling of long term anthropogenic nutrient discharge in China

**DOI:** 10.1038/s41597-024-03102-9

**Published:** 2024-03-09

**Authors:** Haoran Zhang, Huihang Sun, Ruikun Zhao, Yu Tian, Yiming Meng

**Affiliations:** State Key Lab of Urban Water Resource and Environment, School of Environment, Harbin Institute of Technology, Harbin, 150090 China

**Keywords:** Environmental impact, Element cycles, Element cycles, Sustainability

## Abstract

High-resolution integration of large-scale and long-term anthropogenic nutrient discharge data is crucial for understanding the spatiotemporal evolution of pollution and identifying intervention points for pollution mitigation. Here, we establish the MEANS-ST1.0 dataset, which has a high spatiotemporal resolution and encompasses anthropogenic nutrient discharge data collected in China from 1980 to 2020. The dataset includes five components, namely, urban residential, rural residential, industrial, crop farming, and livestock farming, with a spatial resolution of 1 km and a temporal resolution of monthly. The data are available in three formats, namely, GeoTIFF, NetCDF and Excel, catering to GIS users, researchers and policymakers in various application scenarios, such as visualization and modelling. Additionally, rigorous quality control was performed on the dataset, and its reliability was confirmed through cross-scale validation and literature comparisons at the national and regional levels. These data offer valuable insights for further modelling the interactions between humans and the environment and the construction of a digital Earth.

## Background & Summary

Over the past four decades, the biogeochemical cycles of the earth have been profoundly impacted by human activities, with dramatic manifestations in China^1,2^. Notably, from 1980 to 2020, China witnessed substantial increases in fertilizer utilization, livestock farming and population growth, reaching 3.1, 3.7–7.8 and 0.4 times the original levels, respectively. Concomitantly, anthropogenic nitrogen and phosphorus production in China has surged remarkably^3,4^, positioning the nation as one of the major contributors to worldwide anthropogenic nutrient discharge^5^. These changes have escalated nitrogen and phosphorus loads in terrestrial and aquatic systems, resulting in water quality degradation, intensified lake eutrophication, air pollution, stratospheric ozone depletion and global warming^6–8^. In response to these challenges, the Chinese government has highly prioritized pollution control^9–12^, enacting stringent measures that have yielded preliminary success in mitigating nitrogen and phosphorus pollution^13^. Nevertheless, escalating anthropogenic pressure remains a considerable challenge^14,15^. Therefore, assessing the prevailing status and characteristics of long-term anthropogenic nutrient discharge in China is essential for effectively managing chemical nutrient imbalances resulting from human activities and devising appropriate mitigation strategies^16^.

However, the current studies provide only limited insights into the accurate quantification of anthropogenic pollutant discharge for the following three reasons. First, most of the existing accounting systems use fixed or crude parameters that do not adequately express the characteristics of the long-standing and rapidly growing wastewater sector in China, with considerable expansion of the urban and rural wastewater infrastructure and upgrading of the wastewater treatment capacity over the past 40 years^9,17^. Second, there has been a paradigm shift in the lifestyles and production modalities of the populace. For instance, during the rural “Toilet Revolution”, while sanitation for farmers improved, an increase in polluted runoff was concurrently observed. Changes in livestock farming practices also affect pollutant discharge coefficients, which were overlooked in previous studies^18^. Finally, most studies have focused only on one or a few types of pollutant sources, such as crop farming or industrial sources. Simultaneously, studies often concentrate on localized regions^19–21^ and lack consideration of variations in interregional output coefficients^22^, thus limiting a comprehensive understanding of the anthropogenic pollutant discharge pattern in China.

The spatial heterogeneity and temporal nonstationarity of nutrient pollution patterns, exacerbated by anthropogenic disturbances, necessitate the utilization of high-resolution spatiotemporal datasets. Such datasets are instrumental in accurately pinpointing regions that should be prioritized for control measures and capturing the temporal fluctuations in pollution levels^23–25^. The existing large-scale simulations are largely based on administrative units at the provincial or county level and tend to neglect local features and parameters in spatial downscaling or scale conversion. Additionally, there is an increasing reliance on both data-driven and process-based models that utilize high-resolution grid data. For instance, global-scale models such as Global NEWS-2^26^ and national-scale models such as MARINA 1.0^27^, which are designed at the grid or watershed scale rather than the administrative scale, require adaptable downscaling methods to reconcile the ‘variable elements’ issue that emerges from the use of different pollutant source statistics and modelling paradigms. Compounding these challenges, the extant pollutant datasets frequently contain spans of missing values, thus hindering long-term, dynamic and continuous assessments of nutrient trends in China^28^. Moreover, the typically annual temporal resolution of these datasets fails to account for seasonal factors, such as agricultural practices, that significantly influence pollution patterns^29,30^. This mismatch with environmental data scales, including those of meteorological and water quality metrics, further complicates the support for modelling requirements^31,32^. Therefore, the coarseness of the spatial and temporal dimensions of the data is insufficient for comprehensively and finely estimating the environmental impacts caused by pollution, necessitating the development of more refined anthropogenic nutrient discharge datasets with higher spatial and temporal resolutions.

This study presents a “bottom-up” accounting model (MEANS-ST) for tracking anthropogenic pollutant discharge in rivers. It utilizes a spatiotemporal dynamic parameter system consisting of 22 characteristics spanning 40 years. By establishing a comprehensive high-resolution dataset, this model enables a detailed analysis of long-term nutrient discharge from anthropogenic sources. The finding shed light on the factors driving anthropogenic pollutant discharge, providing a holistic understanding of this issue. The monthly scale changes in three typical years, 1980, 2000 and 2020, are tracked at a spatial resolution of 1 km × 1 km. Concomitantly, five major sources were identified, namely, urban residential, rural residential, industrial, crop farming and livestock farming, as were two pollutant types, total nitrogen (TN), and total phosphorus (TP). The research methodology is based on fine-scale and localized spatiotemporally dynamic parameters, combining anthropogenic pollutant discharge accounting methods with spatiotemporal downscaling models. This approach addresses the growing need for precise management measures and bridges the gap between low-resolution discharge inventories and the demand for higher resolution data. Hence, this approach transcends the spatial constraints of “fixed surface” to “arbitrary point” while capturing seasonal variations.

We organize the data in various formats (GeoTIFF, NetCDF and Excel), catering to researchers and policymakers in different disciplines. The dataset can be utilized from various perspectives. First, the dataset reflects the spatial distributions of different pollutants and different types of anthropogenic pollutant sources, revealing the temporal and spatial characteristics of pollutant discharge and supporting the identification of pollution hotspots and patterns. Second, the dataset can be used for the validation of data-based or process-based models in combination with other global data, such as nighttime light and agricultural fertilizer use data, to assess the critical roles of various driving factors in driving changes in anthropogenic pollutant discharge. Third, the dataset serves as a foundation for studies related to environmental management, including setting discharge reduction targets, formulating watershed pollution control strategies, and predicting future pollution loads and water quality levels. Overall, the dataset holds great potential for use in a wide range of applications in pollution control, surface water quality management, river ecology assessment and biodiversity research.

## Methods

**Fig. 1:**
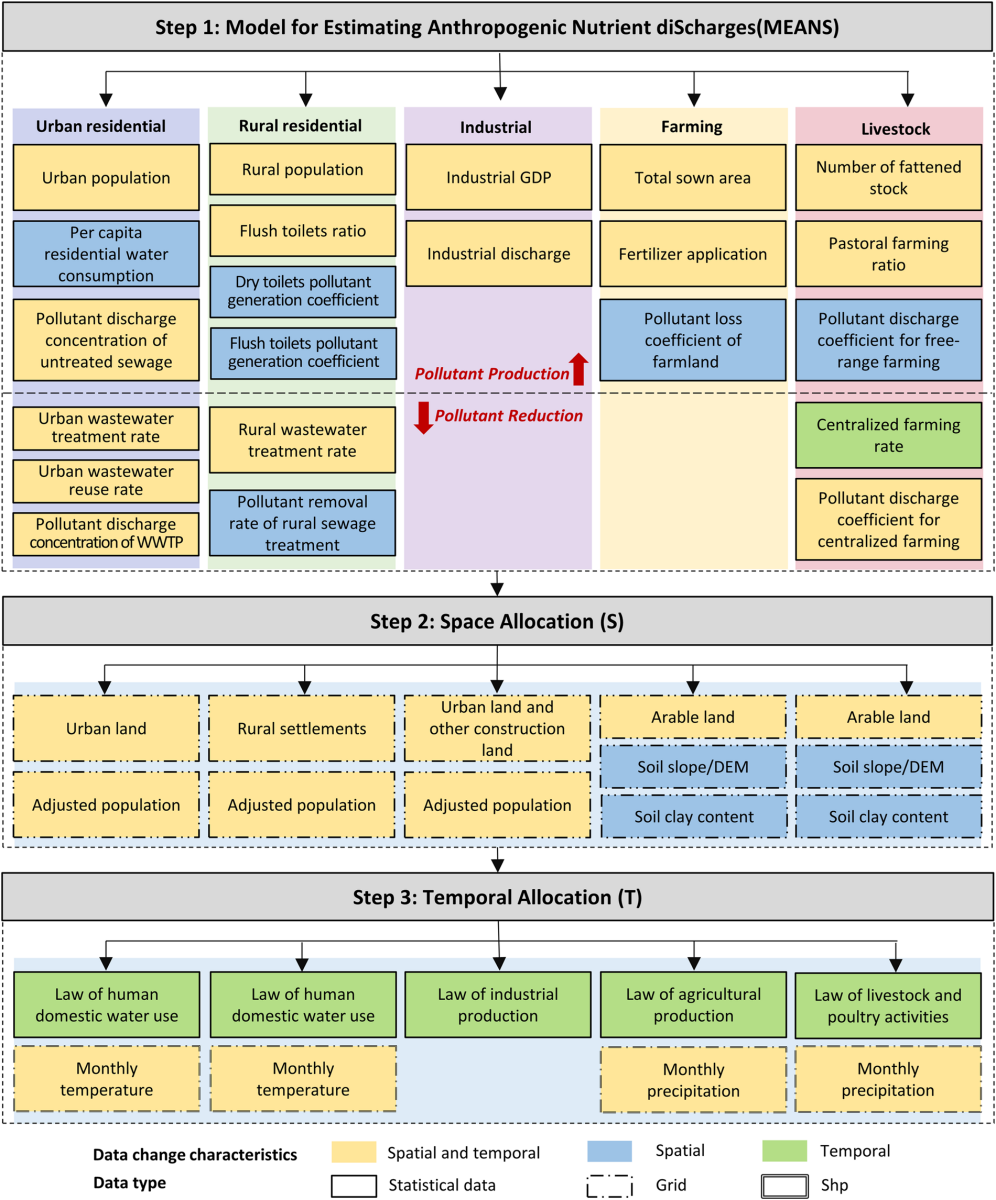
Calculation framework for MEANS-ST1.0. The temporal and spatial data represent parameters that changed during 40 years and between provinces, respectively.

**Fig. 2:**
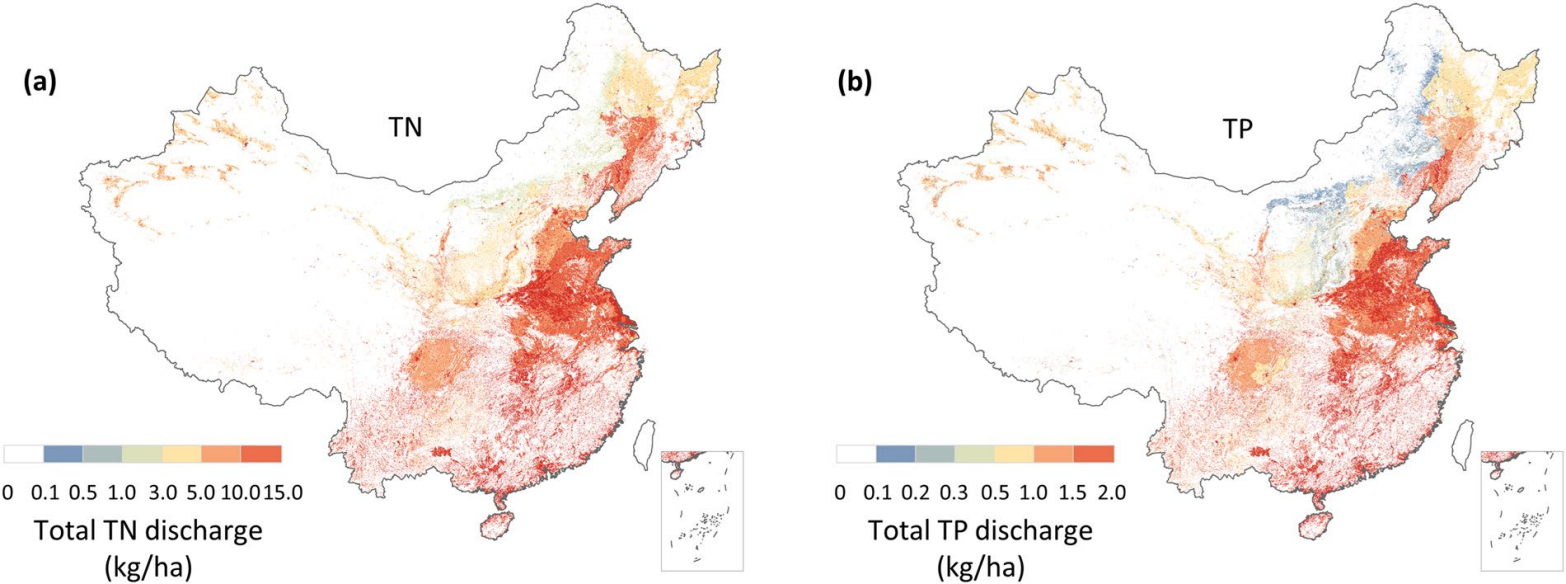
Spatial distribution of China total anthropogenic nutrient discharge in 2020. (**a**) Total anthropogenic TN discharge; (**b**) Total anthropogenic TP discharge. The data of Hong Kong, Macao and Taiwan is absent.

**Fig. 3:**
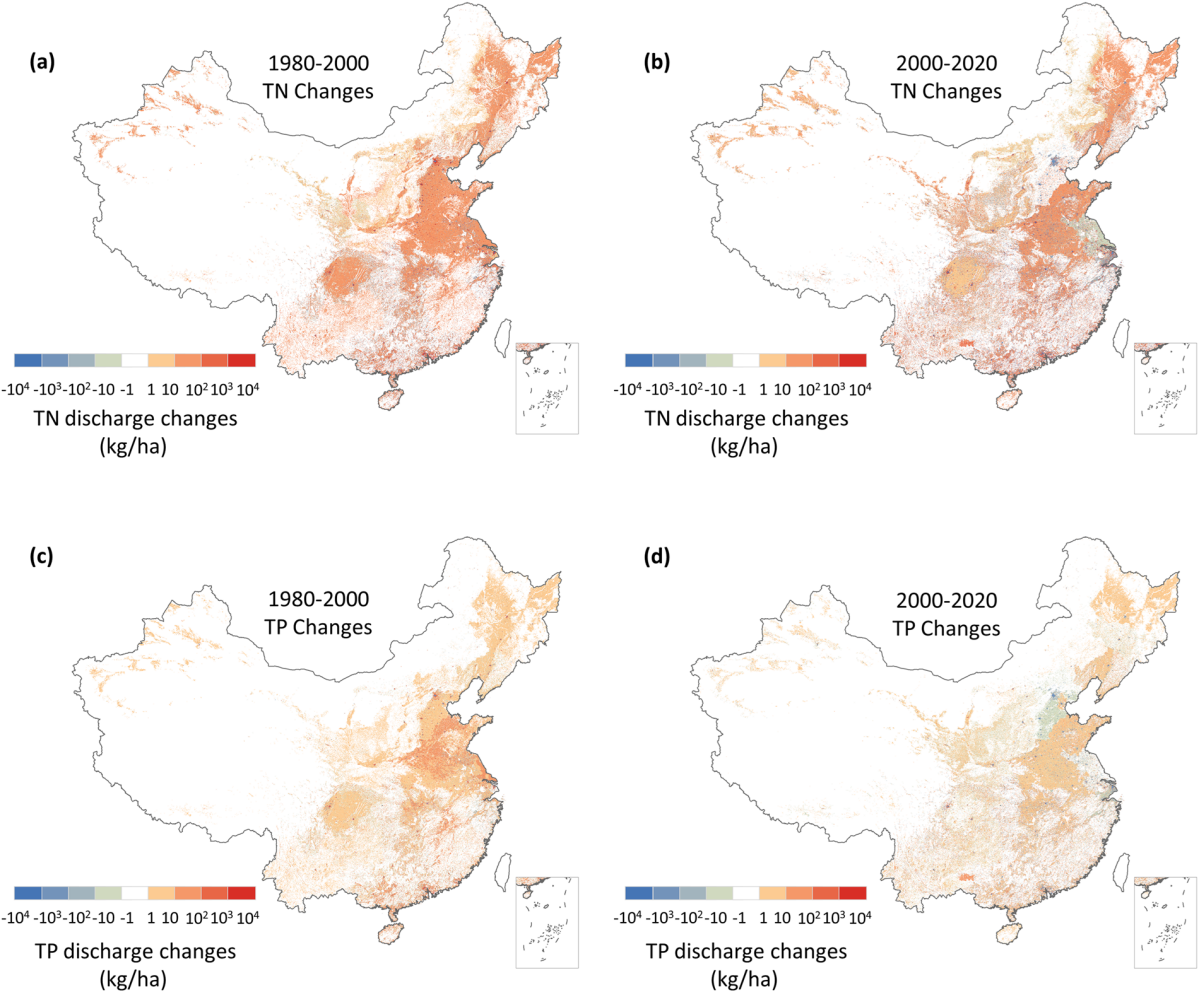
Spatial distribution of changes in total anthropogenic nutrient discharge. (**a**) Changes in total anthropogenic TN discharge from 1980 to 2000; (**b**) Changes in total anthropogenic TN discharge from 2000 to 2020; (**c**) Changes in total anthropogenic TP discharge from 1980 to 2000; (**d**) Changes in total anthropogenic TP discharge from 2000 to 2020. The data of Hong Kong, Macao and Taiwan is absent.

This study presents a novel Model for Estimating Anthropogenic Nutrient diScharge with high Temporal and Spatial resolution dataset (MEANS-ST1.0), which is constructed using the MEANS module coupled with the ST module (Fig. 1). The dataset comprehensively covers five major sectors: urban residential, rural residential, industrial, crop farming and livestock farming. The MEANS module accounts for the long-term dynamic variations in anthropogenic pollutant discharge from 1980 to 2020 at the provincial level in China, the ST module maps the pollutant discharge data from administrative boundaries to grid cells, and pollutant discharge data is transformed from the annual scale to the monthly scale. Moreover, high-resolution maps of TN and TP at a resolution of 1 km × 1 km for the years 1980, 2000, and 2020 are produced (Figs. 2, 3). The hotspot and coldspot distribution are shown in Figure S1 and Table S1. Based on an unprecedented set of 22 spatiotemporal parameters, MEANS-ST1.0 reconstructs the trade-offs between anthropogenic disturbances and environmental support measures to combat long-term anthropogenic pollutant changes in China. As such, this approach provides a detailed and realistic representation of the spatiotemporal features of anthropogenic pollutant discharge. The characteristics and sources of the parameters and geographic data in MEANS-ST1.0 are shown in Tables 1 and S2.

**Table 1:** Sources of geographic data of MEANS-ST1.0.

Descriptions	Data change characteristics	Data type	Data Sources
1:16000000 Province boundary map	Spatial	Shp	Ministry of natural resources. http://bzdt.ch.mnr.gov.cn/^74^
National land use maps of 1 km spatial resolution (1980, 2000, 2020),	Spatial and temporal	Grid (TIFF)	Institute of Geographic Sciences and Natural Resources Research, Chinese Academy of Sciences. https://www.resdc.cn/^75^
Distribution of nine agricultural areas in China	Spatial	Shp	Geographic Data Sharing Infrastructure, Peking University. http://geodata.pku.edu.cn^76^
China gridded population count datasets of 1 km spatial resolution (2000, 2020)	Spatial and temporal	Grid (TIFF)	Global open space population dataset. https://hub.worldpop.org/^77^
China gridded population count datasets of 1 km spatial resolution (1980)	Spatial and temporal	Grid (TIFF)	Shen *et al*.^46^
China digital elevation model data of 1 km spatial resolution	Spatial	Grid (TIFF)	National Cryosphere Desert Data Center. http://www.ncdc.ac.cn/^50^
China soil clay content data of 1 km spatial resolution	Spatial	Grid (TIFF)	Harmonized World Soil Database. https://www.fao.org/soils-portal/en/^51^
China monthly average temperature datasets of 1 km spatial resolution (1980, 2000, 2020)	Spatial and temporal	Grid (NC)	National Earth System Science Data Center. http://www.geodata.cn/^59^
China monthly average precipitation datasets of 1 km spatial resolution (1980, 2000, 2020)	Spatial and temporal	Grid (NC)	National Earth System Science Data Center. http://www.geodata.cn/^59^

### Establishment of the anthropogenic pollutant discharge module

**Fig. 4:**
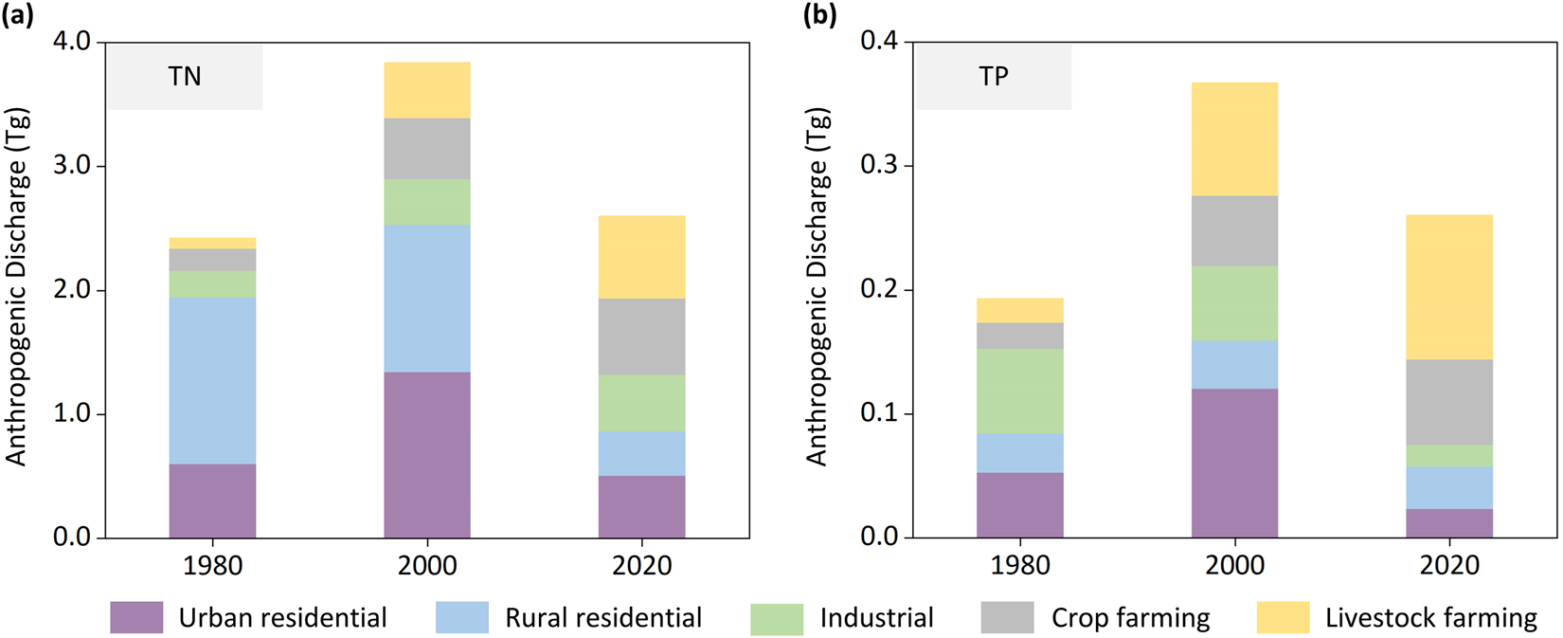
Temporal changes in China total anthropogenic nutrient discharge and their components in 1980, 2000 and 2020. (**a**) Anthropogenic TN discharge; (**b**) Anthropogenic TP discharge.

Anthropogenic pollutant discharge (AD) comprises urban residential pollutant discharge (UD), rural residential pollutant discharge (RD), industrial pollutant discharge (ID), crop farming pollutant discharge (FD), and livestock farming pollutant discharge (LD), as expressed in Eq. (1) (Figs. 4 and S2).

1 $${\rm{AD}}={\rm{UD}}+{\rm{RD}}+{\rm{ID}}+{\rm{FD}}+{\rm{LD}}$$

#### Urban residential anthropogenic pollutant discharge

Within the limits of the MEANS-ST1.0 model, the evaluation of anthropogenic pollution in urban residential areas entails the meticulous quantification of nitrogen and phosphorus discharge generated from routine activities conducted by urban residents. Additionally, this evaluation encompasses the scrutinization of wastewater discharge originating from the service sectors defined in the National Economic Industry Classification (GB/T4754-2017).

Urban residential discharge (UD) consists of two major components, direct discharge (UD_direct_) and discharge after centralized treatment at urban wastewater treatment plants (WWTPs) (UD_treat_), as expressed in Eqs. (2–4). The urban residential population (U_pop_), per capita urban residential water consumption coefficient (UM_water_), proportion of directly discharged water to the total amount of wastewater (UR_direct_), and pollutant concentration in direct wastewater discharge (UC_direct_) are used to calculate the direct discharge component. On the other hand, the centralized treatment component is calculated according to the parameters U_pop_ and UM_water_, the proportion of wastewater treated by WWTPs to the total amount of urban residential wastewater (UR_treat_), the urban wastewater reuse rate (UR_reuse_), and the pollutant concentration in the discharge from WWTPs (UC_treat_).

By incorporating data from the bulletin of the Second National Pollutant Source Census^33^ and considering both temporal and regional variations, we establish provincial-level datasets of direct pollutant concentrations and effluent concentrations from WWTPs in the years 1980, 2000, and 2020. Notably, the effluent concentration dataset is based on the concentration data for WWTP discharge provided by the 2017 Second National Pollutant Source Census, with the average pollutant discharge coefficient for typical plants in different cities used as the provincial discharge coefficient. The data were scaled according to the effluent concentrations at the WWTPs for each year provided in the Report on the Development of Urban Sewage Treatment and Recycling in China^34^ to account for variations over time. Additionally, as one of the 13 countries with the most limited per capita water resources globally^35^, China has shifted its urban wastewater treatment focus from “compliant discharge” to “recycling” since the beginning of the 21st century. By 2020, the nationwide reuse rate of reclaimed water from urban WWTPs had increased from less than 10% in 2010 to 24%^36^. Therefore, wastewater reuse is included in the accounting system, and we establish provincial-level datasets for reuse rates based on the China Statistical Yearbook on the Environment^37^.

2 $${\rm{UD}}={{\rm{UD}}}_{{\rm{direct}}}+{{\rm{UD}}}_{{\rm{treat}}}$$

3 $${{\rm{UD}}}_{{\rm{direct}}}={{\rm{U}}}_{{\rm{pop}}}\times {{\rm{UM}}}_{{\rm{water}}}\times {{\rm{UR}}}_{{\rm{direct}}}\times {{\rm{UC}}}_{{\rm{direct}}}$$

4 $${{\rm{UD}}}_{{\rm{treat}}}={{\rm{U}}}_{{\rm{pop}}}\times {{\rm{UM}}}_{{\rm{water}}}\times {{\rm{UR}}}_{{\rm{treat}}}\times {\rm{(1}}-{{\rm{UR}}}_{{\rm{reuse}}}{\rm{)}}\times {{\rm{UC}}}_{{\rm{treat}}}$$

#### Rural residential anthropogenic pollutant discharge

According to the MEANS-ST1.0 model, the evaluation of anthropogenic pollution in rural residential areas entails quantifying the discharge of nitrogen and phosphorus resulting from the everyday activities of rural residents. This evaluation specifically encompasses the measurement of “grey water” discharge, which is produced by activities such as kitchen tasks, personal hygiene practices, laundry, bathing, and similar actions. Additionally, it involves assessing the discharge from flush toilets, which includes excrement and urine, commonly known as “black water”.

Rural residential discharge (RD) consists of two major components, direct discharge (RD_direct_) and discharge after treatment in rural wastewater treatment facilities (RD_treat_), as expressed in Eqs. (5–8). The urban resident population (R_pop_), the proportion of dry toilets in rural areas (RR_dry_), the proportion of flush toilets in rural areas (RR_flu_), the pollutant generation coefficient of rural residents using dry toilets (RM_dry_), the pollutant generation coefficient of rural residents using flush toilets (RM_flu_), and the proportion of direct wastewater to the total wastewater volume in rural areas (RM_direct_) are used to calculate direct discharge. On the other hand, the treatment component is calculated according to the above parameters and the proportion of treated wastewater to the total wastewater volume in rural areas (RM_treat_) and the pollutant removal rate of rural wastewater treatment (RM_removal_).

In recent years, China has vigorously promoted the improvement of rural living environments, with rural toilet pollution control being an important aspect^38^. According to statistics, the N and P contents in toilet wastewater account for 86.3% and 80.5%, respectively, of the total pollutants in rural residential wastewater. Additionally, the per capita discharge coefficient of dry toilets is significantly lower than that of flush toilets^39^. Therefore, we distinguish between different types of toilets in our analysis. Since rural wastewater treatment in China started relatively late, we consider regional differences in the pollutant removal efficiency of different rural treatment facilities but do not consider temporal changes in this study. Provincial and annual datasets of wastewater treatment incidence and toilet modality ratios are formulated based on data from the China Urban‒Rural Construction Statistical Yearbook^40^ and the China Statistical Yearbook on the Environment^37^. The pollutant generation coefficients and pollutant removal efficiencies are derived from the Second National Pollutant Source Census coefficient manuals.

5 $${\rm{RD}}={{\rm{RD}}}_{{\rm{direct}}}+{{\rm{RD}}}_{{\rm{treat}}}$$

6 $${{\rm{RR}}}_{{\rm{generation}}}={{\rm{RR}}}_{{\rm{dry}}}\times {{\rm{RM}}}_{{\rm{dry}}}+{{\rm{RR}}}_{{\rm{flu}}}\times {{\rm{RM}}}_{{\rm{flu}}}$$

7 $${{\rm{RD}}}_{{\rm{direct}}}={{\rm{R}}}_{{\rm{pop}}}\times {{\rm{RR}}}_{{\rm{generation}}}\times {{\rm{RM}}}_{{\rm{direct}}}$$

8 $${{\rm{RD}}}_{{\rm{treat}}}={{\rm{R}}}_{{\rm{pop}}}\times {{\rm{RR}}}_{{\rm{generation}}}\times {{\rm{RM}}}_{{\rm{treat}}}\times {\rm{(1}}-{{\rm{RM}}}_{{\rm{removal}}}{\rm{)}}$$

#### Industrial anthropogenic pollutant discharge

In the MEANS-ST1.0 model, industrial anthropogenic pollution can be assessed, mainly involving the meticulous estimation of nitrogen and phosphorus discharge from a wide range of industrial enterprises operating in the mining, manufacturing, electricity generation, heat production, gas supply, and water production and supply sectors. These industries are closely aligned with the National Economic Industry Classification (GB/T4754-2017), which includes a total of 41 different comprehensive industrial categories strategically distributed across three main sectors.

Drawing from the China Statistical Yearbook on the Environment and the Statistical Yearbooks of various provinces, we establish a provincial annual dataset of industrial pollutant discharge (ID). Simultaneously, we develop functional relationships between different industrial pollutant discharge forms and GDP for 31 provinces from 1980 to 2020 and extrapolate data for missing years (Figs. S3, S4).

#### Crop farming anthropogenic pollutant discharge

Assessing crop farming anthropogenic pollution, as addressed in the MEANS-ST1.0 model, encompasses the meticulous quantification of nitrogen and phosphorus movement from soil and fertilizers into the water environment during precipitation and irrigation events in the cultivation of various crops, such as grains, cash crops, fruits, and vegetables.

As expressed in Eqs. (9–10), employing the pollutant loss coefficient (FM_2017_) provided in the Second National Pollutant Source Census conducted in 2017 as the baseline, we correct these values in accordance with changes in fertilizer application rates to obtain a temporal dataset of farmland pollutant loss. The farmland sown area (F_area_) and farmland fertilizer application (F_fertizer, i_) data were obtained from national and provincial statistical yearbooks^41^.

9 $${\rm{FD}}={{\rm{F}}}_{{\rm{area}}}\times {{\rm{FM}}}_{{\rm{i}}}$$

10 $${{\rm{FM}}}_{{\rm{i}}}={{\rm{FM}}}_{{\rm{2017}}}\times \frac{{{\rm{F}}}_{{\rm{fertilizer,i}}}}{{{\rm{F}}}_{{\rm{fertilizer,2017}}}}$$

#### Livestock farming anthropogenic pollutant discharge

The MEANS-ST1.0 model incorporates a comprehensive evaluation of livestock farming anthropogenic pollution and entails the precise measurement of nitrogen and phosphorus discharge originating from both centralized farming and free-range farming. This evaluation specifically accounts for the proportion of untreated primary pollutants emitted by livestock in six distinct categories, namely, pigs, dairy cows, beef cattle, laying hens, broiler chickens, and sheep. These discharge types are mitigated through treatment facilities or directly discharged into the water environment without undergoing any form of treatment or utilization.

Livestock farming discharge (LD) consists of two major components, namely, centralized (LD_centralized_) and free-range (LD_free_), as expressed in Eqs. (11–13). Here, L_num_ is the number of livestock (i=1, 2, 3, 4, 5, and 6, corresponding to six types of livestock: pigs, beef cattle, dairy cows, laying hens, broilers and sheep, respectively). The centralized portion is calculated according to the centralized farming rate (LR_centralized,i_) and the pollutant discharge coefficient for centralized farming (LM_centralized,i_). Additionally, the free-range portion is calculated based on the free-range farming rate (LR_free,i_) and the pollutant discharge coefficient for free-range farming (LM_free,i_). Notably, we use the pastoral farming ratio (LR_pst,i_) to adjust the livestock quantities of beef cattle, dairy cattle, and sheep, and the portions of pollutants that infiltrate grassland soils are removed.

We address discharge from livestock farming, acknowledging its significant role as a source of agricultural nonpoint surface pollution in China. Notably, 25–30% of livestock manure is estimated to enter water bodies^42^. Thus, livestock farming pollution is one of the most significant sources of agricultural nonpoint surface pollution in China^43,44^. For livestock farming pollutant discharge, we consider a total of six major livestock species: pigs, beef cattle, dairy cows, laying hens, broilers and sheep. Moreover, we distinguish among farming regions (pastoral and nonpastoral areas) and farming methods (centralized farming and free-range farming). Aquaculture is neglected in the manuscript because it accounts for only 1% of the total anthropogenic pollutant discharge from livestock farming^6^. The annual dataset for determining the proportion of pastoral farming and the proportion of centralized farming is from the China Animal Husbandry and Veterinary Yearbook^45^, and the discharge coefficients for different livestock categories are obtained from the Second National Pollutant Source Census manuals and literature studies.

11 $${\rm{LD}}={{\rm{LD}}}_{{\rm{centralized}}}+{{\rm{LD}}}_{{\rm{free}}}$$

12 $${{\rm{LD}}}_{{\rm{centralized}}}={\sum }_{{\rm{i}}=1}^{{\rm{6}}}{{\rm{L}}}_{{\rm{num}}}\times {\rm{(}}1-{{\rm{LR}}}_{{\rm{pst,i}}}{\rm{)}}\times {{\rm{LR}}}_{{\rm{centralized,i}}}\times {{\rm{LM}}}_{{\rm{centralized,i}}}$$

13 $${{\rm{LD}}}_{{\rm{free}}}={\sum }_{{\rm{i}}=1}^{{\rm{6}}}{{\rm{L}}}_{{\rm{num}}}\times {\rm{(}}1-{{\rm{LR}}}_{{\rm{pst,i}}}{\rm{)}}\times {{\rm{LR}}}_{{\rm{free,i}}}\times {{\rm{LM}}}_{{\rm{free,i}}}$$

### Spatial allocation module

#### Population calibration

Population data collected at the administrative unit level may not align with natural boundaries, leading to the “modifiable areal unit problem”. Additionally, representing population information using the average density in administrative units fails to capture the fine-scale spatial distribution characteristics of the population and may hinder the visualization and exploration of population distribution patterns. To address these issues, we integrate provincial-level population data from the Fifth National Population Census (2000) and the Seventh National Population Census (2020) of China with provincial-level population statistics from the 1980 statistical yearbooks. This integration facilitates the creation of a calibrated population dataset for representative years in China.

We define the population distribution at a spatial resolution of 1 km as the reference population distribution. The data for the year 1980 are obtained from the China Population Geographic Distribution dataset developed by Shen *et al*.^46^, and the data for 2000 and 2020 are obtained from the Worldpop Global Population Distribution dataset. The population distribution within each administrative region is assumed to be geographically proportional to the reference population distribution according to Eqs. (14–15). This step is implemented using ESRI ArcGIS 10.7 software.

14 $${{\rm{GRIDPOP}}}_{{\rm{i,y}}}={{\rm{POPC}}}_{{\rm{c,y}}}\times {{\rm{RGRIDPOP}}}_{{\rm{i,y}}}$$

15 $${{\rm{POPC}}}_{{\rm{c,y}}}=\frac{{{\rm{STAPOP}}}_{{\rm{c}}}}{{{\rm{RPOP}}}_{{\rm{c}}}}$$

where i is the specified grid, y represents the year, GRIDPOP_i,y_ is the population in the ith grid in year y, and subscript c is the province where the i^th^ grid is located. POPC_c,y_ is the population correction factor for province c in year y, and RGRIDPOP_i,y_ is the population in the ith grid in year y, as the reference population. STAPOP_c_ denotes the statistical population of province c, and RPOP_c_ denotes the population based on the reference map of province c.

#### Spatial allocation

**Fig. 5:**
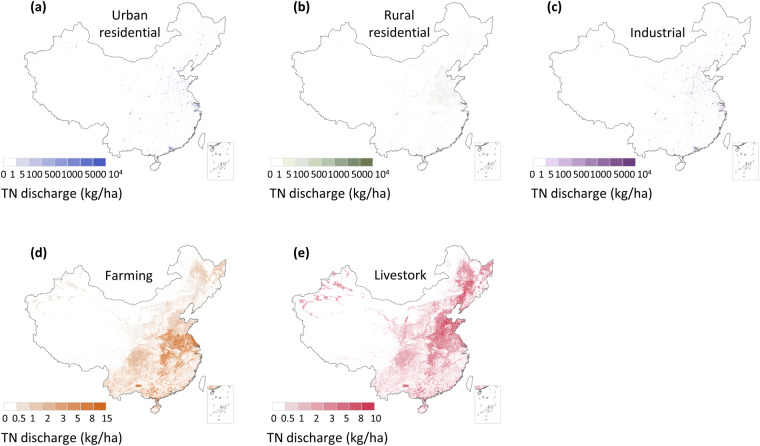
Spatial distribution of different components of anthropogenic TN discharge in 2020. (**a**) Urban residential pollutant discharge to urban land; (**b**) Rural residential pollutant discharge to rural residential land; (**c**) Industrial pollutant discharge to urban land and other construction land; (**d**) Crop farming pollutant discharge to cultivated land; (**e**) Livestock farming pollutant discharge to cultivated land. The data of Hong Kong, Macao and Taiwan is absent.

**Fig. 6:**
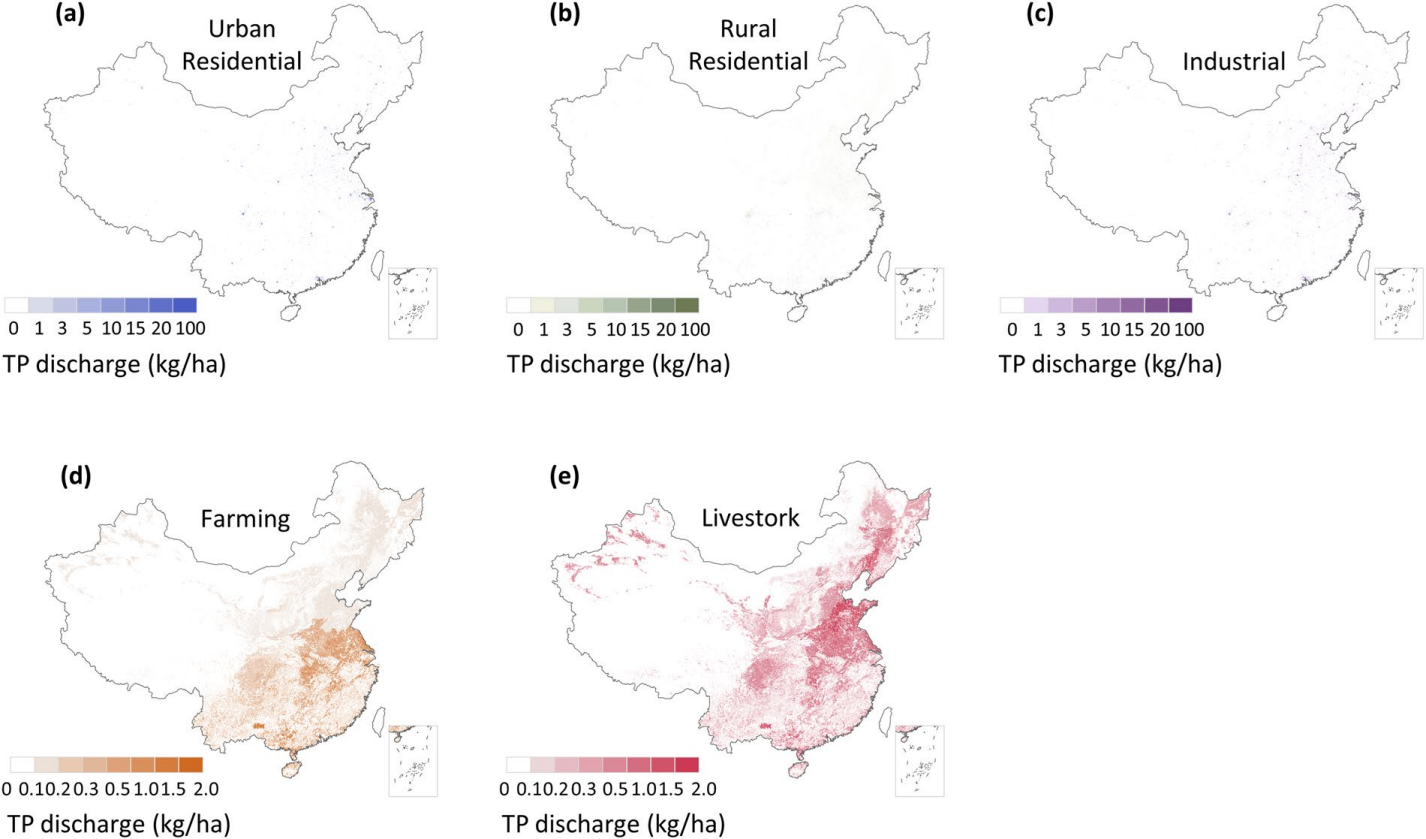
Spatial distribution of different components of anthropogenic TP discharge in 2020. (**a**) Urban residential pollutant discharge to urban land; (**b**) Rural residential pollutant discharge to rural residential land; (**c**) Industrial pollutant discharge to urban land and other construction land; (**d**) Crop farming pollutant discharge to cultivated land; (**e**) Livestock farming pollutant discharge to cultivated land. The data of Hong Kong, Macao and Taiwan is absent.

Building upon the MEANS dataset, we develop a spatial allocation model and propose a method for downscaling coarse-resolution data available at the regional or national level to provide fine-resolution data for each grid cell (Figs. 5, 6). The spatial downscaling of each sector is conducted independently and is achieved through the following steps.

First, we utilize national land use type data with a spatial resolution of 1 km for the years 1980, 2000 and 2020. By employing a land use region proportion-sharing approach, we disaggregate the data for the provincial variable of anthropogenic discharge into 1 km grid units in the Krasovsky_1940_Albers projection spatial reference system based on Eq. (16)^44^. Specifically, urban land is associated with urban residential and industrial pollutant discharge types, rural residential land is linked to rural residential pollutant discharge, other constructed land is associated with industrial pollutant discharge, and cultivated land (including paddy fields and drylands) is linked to crop farming and livestock farming pollutant discharge types (Table 2). Subsequently, as the production of residential and industrial pollution is strongly correlated with the population distribution at the regional level, we utilize population data as a spatial surrogate to indicate the proportion of pollutant discharge allocated to each grid cell.

16 $${{\rm{LandUse}}}_{{\rm{i,k,t}}}=\left\{\begin{array}{c}{\rm{1,}}\,{\rm{if}}\,{\rm{i}}\,{\rm{belongs}}\,{\rm{to}}\,{\rm{land}}\,{\rm{use}}\,{\rm{type}}\,{\rm{k}}\\ {\rm{0,}}\,{\rm{others}}\end{array}\right.$$

where i is the number of grid cells and k denotes the land use type at time t, which represents the year. LandUse_i,k,t_ indicates whether grid i belongs to land use type k in year t.

**Table 2:** Land use categories attributed to different pollutant-producing sectors.

Anthropogenic Pollutant Discharge	Land Use Type
Urban residential discharge	Urban land
Rural residential discharge	Rural residential land
Industrial discharge	Urban land, other construction land
Crop farming discharge	Cultivated land
Livestock farming discharge	Cultivated land

Dong, L. *et al*.’s study^47^ demonstrated that soil nutrient loss varies based on factors like slope and soil type. Shi, W.^48^ found that steeper slopes directly correlate with higher slope soil instability, especially on heavily disturbed farmland. Huo, J. *et al*.^49^, in their investigation of loess hilly areas, discovered a significant positive correlation between clay content and soil nutrient levels, highlighting the role of clay particles in nutrient enrichment. To represent agricultural sources, slope and soil clay content were selected as spatial surrogates. The study utilized 1 km resolution digital elevation model data from the National Cryosphere Desert Data Center^50^ and clay content data from the Harmonized World Soil Database (HWSD)^51^ to determine nutrient loss coefficient grades for various slope and clay content levels (Tables 3, 4)^52,53^. The study considered the spatial correlation between agricultural nutrient discharge and proxy indicators such as slope and soil clay content by assigning relevant coefficients at the spatial scale.

**Table 3:** Grade classification of nutrient loss coefficients based on slope.

Slope	Loss Coefficient
Horizontal (0–8% slope)	0.10
Moderate (8–15% slope)	0.20
Medium (15–25% slope)	0.35
Steep (>25% slope)	0.50

**Table 4:** Grade classification of nutrient loss coefficients based on soil clay content.

Clay Content	Loss Coefficient
Coarse (0–18% clay)	0.75
Medium (18–35% clay)	0.50
Fine (35–60% clay)	0.25
Very Fine (>60% clay)	0.10

### Temporal allocation module

**Fig. 7:**
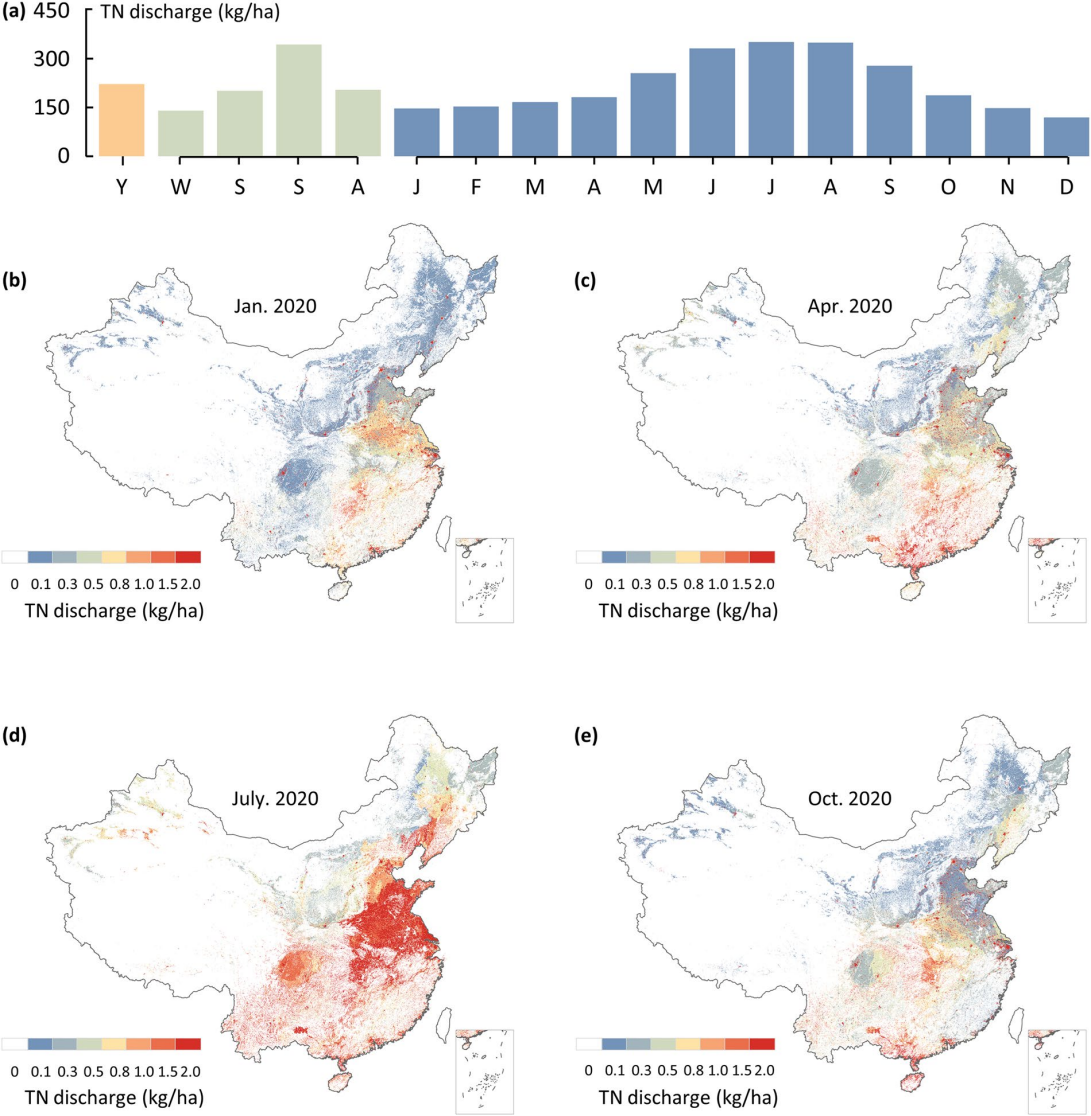
Temporal profile of total anthropogenic TN discharge and spatial distribution of typical months in 2020. (**a**) Yearly(mean), monthly and seasonally weights for total anthropogenic TN discharge in 2020; (**b–e**) Spatial distribution of total anthropogenic TN discharge in January, April, July and October in 2020. The data of Hong Kong, Macao and Taiwan is absent.

**Fig. 8:**
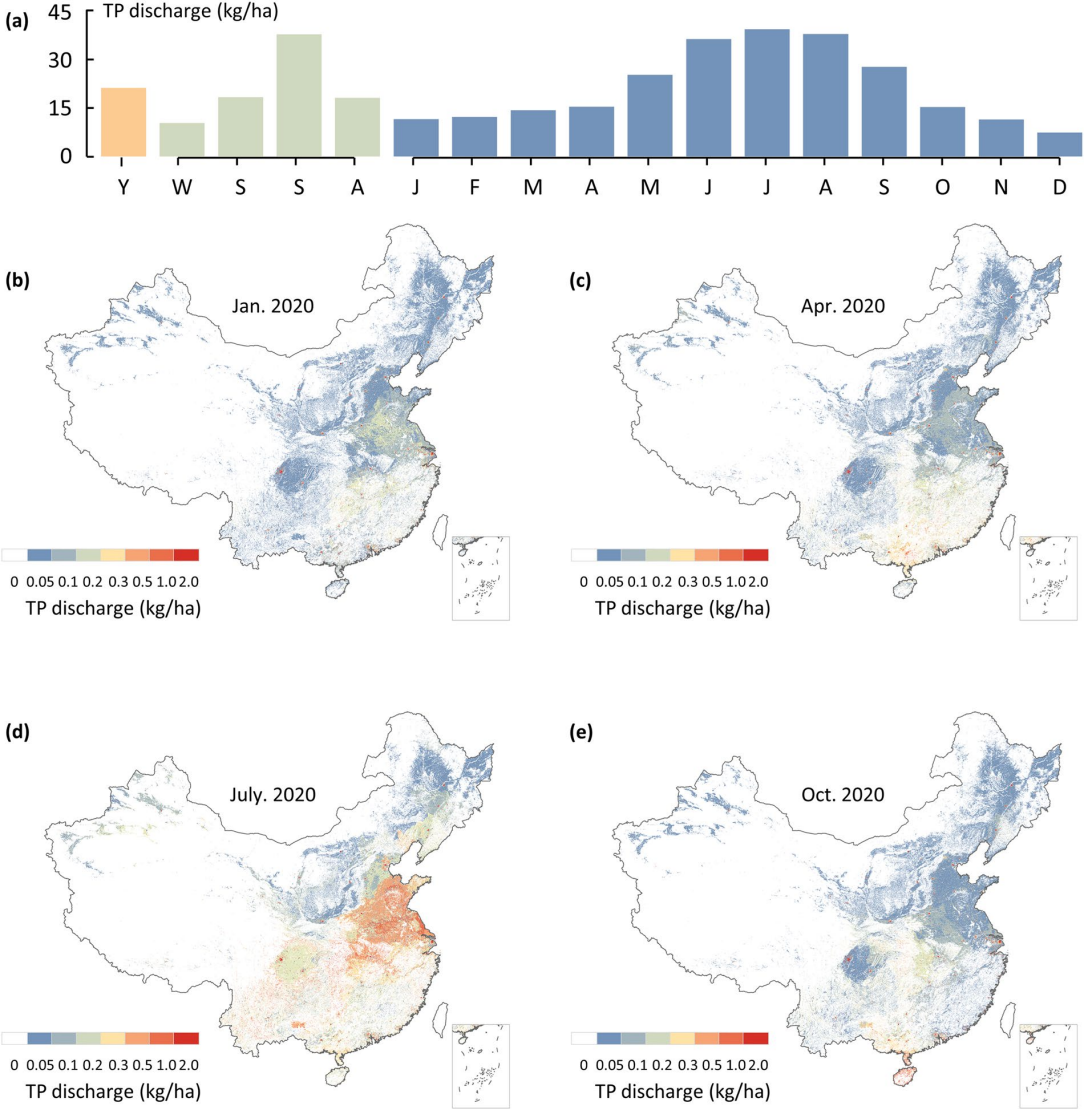
Temporal profile of total anthropogenic TP discharge and spatial distribution of typical months in 2020. (**a**) Yearly(mean), monthly and seasonally weights for total anthropogenic TP discharge in 2020; (**b-e**) Spatial distribution of total anthropogenic TP discharge in January, April, July and October in 2020. The data of Hong Kong, Macao and Taiwan is absent.

Human activities are influenced by temperature and seasonality, and agricultural pollution discharge exhibit significant seasonality due to the flushing effect of rainfall, making it difficult to support seasonal analyses of anthropogenic pollutant discharge data at an annual scale. To address this issue, a set of techniques is employed to refine annual values to monthly values (Figs. 7, 8). Temporal downscaling for each sector is independently executed based on the following steps. First, we calculate allocation coefficients for each sector in different months. Second, the “Raster Calculator” module in the spatial analysis toolbox in ESRI ArcGIS 10.7 is used to perform algebraic operations on high-resolution, annual-scale maps.

For residential sources, the generation of pollutants is related to water consumption and the amount of wastewater containing pollutants. The composition of wastewater does not usually exhibit seasonality, while water consumption varies across different months due to temperature effects^54^. Drawing on the methodology advanced by N. Voisin *et al*.^55^, who crafted water consumption maps at a monthly resolution, we adopt a parallel strategy for assessing urban and rural residential sources to achieve monthly scale allocation, as expressed in Eqs. (17–19).

17 $${{\rm{C}}}_{{\rm{i,j}}}=\frac{1}{12}\left(\frac{{{T}}_{{\rm{i,j}}}-{{T}}_{\mathrm{avg}{\rm{j}}}}{{{T}}_{\max {\rm{j}}}-{{T}}_{\min {\rm{j}}}}{R}+{\rm{1}}\right)$$

18 $${{\rm{UD}}}_{{\rm{i,j}}}={{\rm{UD}}}_{{\rm{j}}}\times {{\rm{C}}}_{{\rm{i,j}}}$$

19 $${{\rm{RD}}}_{{\rm{i,j}}}={{\rm{RD}}}_{{\rm{j}}}\times {{\rm{C}}}_{{\rm{i,j}}}$$

where C_i, j_ represents the residential source allocation coefficient for month i and year j; T_i, j_ denotes the monthly temperature; and T_avg j_, T_max j_, and T_min j_ represent the average, maximum, and minimum temperatures of the year, respectively. R is the amplitude (dimensionless), which denotes the relative difference in residential water use between the warmest and coldest months of the year. Huang *et al*. suggested a value of 0.2 for R based on an assessment of China^56^.

For agricultural sources, the discharge of pollutants is related to the leaching of fertilizers applied during crop cultivation. Since different crops have distinct fertilization schedules, the monthly allocation of agricultural cultivation source loads should consider the types of crops and the fertilization time. Furthermore, rainfall exacerbates the effects of nutrient loss, resulting in pronounced seasonal dynamics and interannual variability in agricultural pollutant loads^57,58^. Using vector data for the nine major agricultural regions in China, we identify typical crops and the corresponding fertilizer application conditions in different agricultural regions considering the established patterns of agricultural production. Additionally, we utilized the 1 km monthly average rainfall dataset for China^59^ (http://www.geodata.cn/). This method facilitates the monthly scale allocation of agricultural sources according to Eqs. (20–21).

20 $${{\rm{FC}}}_{{\rm{i,j}}}=\frac{{{\rm{P}}}_{{\rm{i,j}}}}{{{\rm{P}}}_{{\rm{avg}}{\rm{j}}}}\times {\sum }_{{\rm{i}}={\rm{1}}}^{{\rm{n}}}\left({{\rm{A}}}_{{\rm{n,i}}}\times {{\rm{F}}}_{{\rm{n,i}}}\right)$$

21 $${{\rm{FD}}}_{{\rm{i,j}}}={{\rm{FD}}}_{{\rm{j}}}\times {{\rm{FC}}}_{{\rm{i,j}}}$$

where FC_i, j_ represents the agricultural source allocation coefficient for month i and year j, P_i, j_ denotes the monthly rainfall; and T_avg j_, represent the average rainfall of the year, A_n, i_ is the coefficient of the proportion of the annual sown area of the n^th^ crop to the total sown area of the crop in the agricultural area in month i, and F_n, i_ is the coefficient of the proportion of fertilizer applied to the n^th^ crop to the annual application of the fertilizer in month i.

For livestock farming, discharge shows a clear diurnal pattern influenced by feeding, drinking, and resting times^60^. Additionally, the discharge of pollution from livestock farming exhibits a certain level of seasonality due to the flushing effect of rainfall. The 1 km monthly average rainfall dataset for China^59^ (http://www.geodata.cn/) was used to establish a temporal correlation between historical rainfall and discharge from livestock farming, enabling monthly-scale allocation of livestock sources according to Eqs. (22–23).

22 $${{\rm{LC}}}_{{\rm{i,j}}}=\frac{{{\rm{P}}}_{{\rm{i,j}}}}{{{\rm{P}}}_{{\rm{avg}}{\rm{j}}}}$$

23 $${{\rm{LD}}}_{{\rm{i,j}}}={{\rm{LD}}}_{{\rm{j}}}\times {{\rm{LC}}}_{{\rm{i,j}}}$$

where LC_i, j_ represents the livestock farming source allocation coefficient for month i and year j, P_i, j_ denotes the monthly precipitation; and T_avgj_, represent the average precipitation of the year.

Unlike residential and agricultural sources, most industrial pollution discharge does not exhibit significant differences among seasons. Therefore, the annual discharge of industrial pollutant is evenly distributed across each month to achieve monthly-scale allocation according to Eq. 24.

24 $${{\rm{ID}}}_{{\rm{i,j}}}={{\rm{ID}}}_{{\rm{j}}}\times \frac{1}{12}$$

## Data Records

The MEANS-ST1.0 dataset consists of a “Data File” and a “Readme File”, which are freely available on the Figshare^61^. The “Data File” serve as the core file, while the “Readme File” provides explanations of abbreviations and units, along with a list of key parameters (Tables S3–S8). Within the data files, we offer three different formats of anthropogenic pollutant discharge datasets. The first format is stored as GeoTIFF files, which can be used in conjunction with GIS software for overall characterization and spatial distribution analysis. The spatial resolution is 1 km, covering three representative years (1980, 2000, and 2020) and providing data on total anthropogenic nitrogen discharge, as well as discharge from five types of anthropogenic pollutant sources: urban residential, rural residential, industry, crop farming and livestock farming. The second format comprises ten NetCDF files, suitable for constructing two-dimensional or multi-dimensional models and conducting data visualization analysis. These files have a spatial resolution of 1 km and contain monthly data for different years (1980, 2000, and 2020) on total TN and TP discharge and five types of anthropogenic pollutant sources. The third format of the dataset is Excel files, supporting the construction of a national integrated model and providing yearly data on anthropogenic pollutant discharge for provincial administrative units, including both total and categorized discharge. The MEANS-ST1.0 dataset incorporates the most comprehensive spatiotemporal dynamic parameters, enabling a fine-grained analysis of the long-term dynamics for China’s anthropogenic nutrient discharge from both spatial and temporal perspectives.

## Technical Validation

### Quality control of the dataset

Anthropogenic pollutant discharge is a long-term characteristic of economic and social development, and the associated processes are challenging to validate through experiments. Therefore, this study involves highly data-intensive modelling, with strict data quality assurance and control (QA/QC) to ensure the reliability of model outputs^62^. Qualitative and quantitative quality assessments of each parameter in the MEANS-ST1.0 dataset are presented in Table 5. We conduct thorough QA/QC on the dataset from six dimensions: data collection, data independence, data representation, data age, geographical relevance, and technological relevance (Table S9). Low values of evaluation indicators indicate better data quality from their respective perspectives. Moreover, the dataset is meticulously examined to identify possible errors and outliers using domain-specific knowledge and expertise, ensuring the reliability of the data.

**Table 5:** Results of the data quality assessment.

Category	DAQ	DI	DR	DA	GC	TC	Average value
U_pop_	1	1	1	1	1	1	1.0
UM_water_	1	1	3	2	1	1	1.5
UR_treat_	1	1	1	1	1	1	1.0
UR_reuse_	1	1	1	1	1	1	1.0
UC_direct_	1	1	1	1	1	1	1.0
UC_treat_	2	3	1	1	1	1	1.5
R_pop_	1	1	1	1	1	1	1.0
RR_flu_	1	1	1	1	1	1	1.0
RM_dry_	1	1	3	3	1	1	1.7
RM_flu_	1	1	3	3	1	1	1.7
RM_treat_	1	1	1	1	1	1	1.0
RM_removal_	1	1	3	3	1	1	1.7
ID	3	3	1	1	1	1	1.7
GDP	1	1	1	1	1	1	1.0
F_area_	1	1	1	1	1	1	1.0
F_fertilizer_	1	1	1	1	1	1	1.0
FM	2	3	3	3	1	1	2.2
L_num_	1	1	1	1	1	1	1.0
LR_centralized_	1	1	2	1	1	1	1.2
LM_centralized_	1	1	3	3	1	1	1.7
LM_free_	1	1	3	3	1	1	1.7
LR_pastoral_	1	1	3	2	1	1	1.5

All the data in the MEANS-ST1.0 database originate from the following officially published national and provincial statistical yearbooks: the China Statistical Yearbook, the China Statistical Yearbook on the Environment, the China Animal Husbandry and Veterinary Yearbook, and the National Pollutant Source Census. Simultaneously, the high-resolution maps (e.g., land use and population data) used in the dataset are obtained from domestic and international high-quality authoritative databases. Additionally, the latest research findings from the referenced literature are used to calibrate certain model parameters (e.g., monthly differences in residential water consumption) to improve accuracy. Overall, this study provides high-quality estimates of anthropogenic pollutant discharge in China under existing conditions.

### Cross-scale validation of the mapping data

**Fig. 9:**
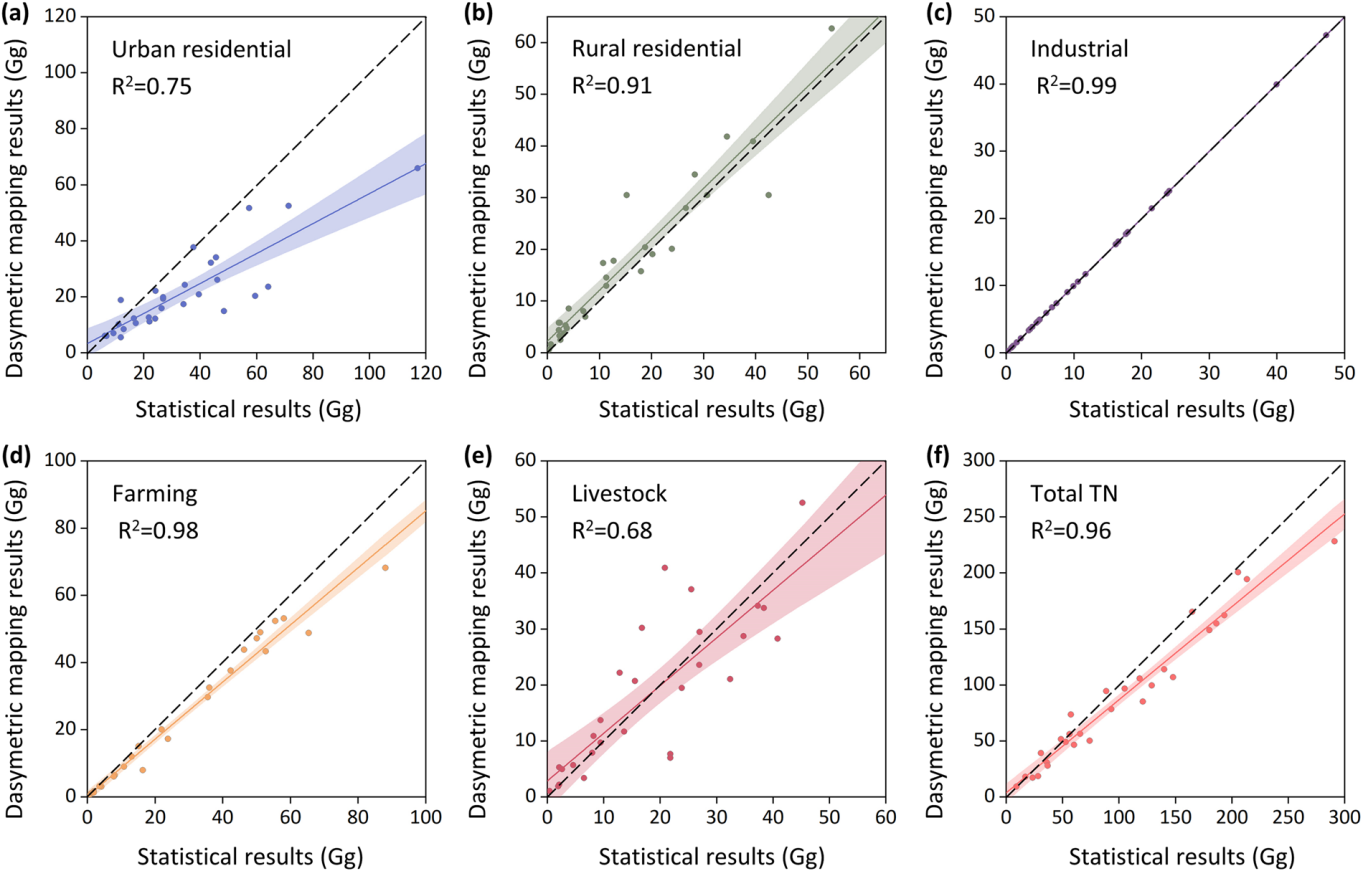
Cross-scale validation for anthropogenic pollutant discharge of TN. (**a**–**e**) Cross-scale validation for urban residential pollutant discharge, rural residential pollutant discharge, industrial pollutant discharge, crop farming pollutant discharge and livestock farming pollutant discharge; (**f**) Cross-scale validation for total pollutant discharge.

**Fig. 10:**
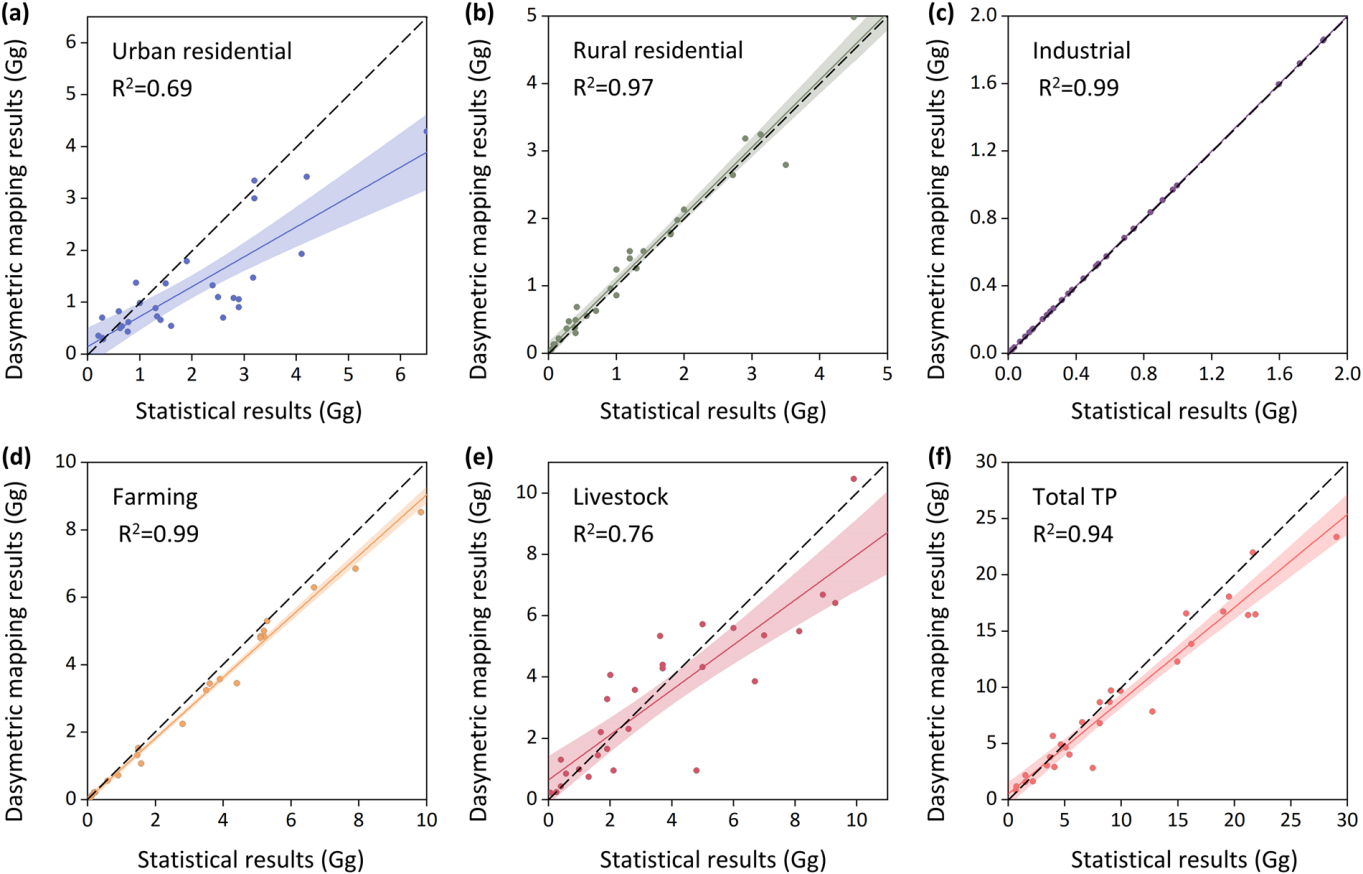
Cross-scale validation for anthropogenic pollutant discharge of TP. (**a**-**e**) Cross-scale validation for urban residential pollutant discharge, rural residential pollutant discharge, industrial pollutant discharge, crop farming pollutant discharge and livestock farming pollutant discharge; (**f**) Cross-scale validation for total pollutant discharge.

The Second National Pollutant Source Census data released in 2017 are the most authoritative, extensive and statistically complete type of pollutant discharge data in China to date. To validate the accuracy of the MEANS-ST1.0 dataset at different scales, we compared the high-resolution mapping results of provincial administrative units in 2017 with the Second National Pollutant Source Census data from all provinces. Figures 9, 10f show a comparison of the results of total pollutant discharge, and both figures indicate good performance, with the data points clustered around the 1:1 diagonal line and fitting coefficients (R^2^) of 0.96 and 0.94, respectively. Figures 9, 10(a–e) present comparisons of results for different pollutant sources. The verification R^2^ for the industrial discharge of both pollutants is 0.99, while for rural residential discharge and crop farming discharge, the R^2^ exceeds 0.9. Simultaneously, the R^2^ for urban residential and livestock farming discharge is above 0.68. These results demonstrate the similarity between the MEANS-ST1.0 dataset and the existing national and provincial census data.

Notably, industrial anthropogenic pollutant discharge characteristics are directly calculated based on national statistical data, resulting in lower uncertainty than that associated with other pollutant discharge types. For urban residential discharge, the calculation of pollutant discharge relies heavily on the pollutant concentration in wastewater discharged from WWTPs, as most provinces achieved high levels of wastewater treatment in 2017. Due to the lack of operational data for urban WWTPs, we use the average pollutant discharge coefficient from typical WWTPs in the Second National Pollutant Source Census as the discharge coefficient for each province, which may introduce some uncertainties into the results^63^. In response to evolving management policies for livestock farming, China has initiated a series of strategic interventions, notably the “relocation of pig farms” initiative. This policy has catalysed the progressive migration of pig farms away from population-dense residential areas to potential areas with abundant feed resources^64^. These measures have influenced the evolution of the pattern of livestock farming pollution in China to some extent. The decadal national pollutant source census may not fully capture the changes in livestock farming nutrient discharge in recent years. However, within the framework of MEANS-ST1.0, the impacts of these policies have been adequately considered.

### Comparative analysis with literature studies

We compare the results based on the MEANS-ST1.0 dataset with those of other modelling studies to demonstrate the reliability of the dataset (Table S10). At a national scale, G. Van Drecht *et al*. estimated global urban wastewater nitrogen and phosphorus discharge for the period of 1970–2050 (IMAGE); the total anthropogenic nitrogen and phosphorus discharge in China was 4.1 Tg and 0.6 Tg, respectively^65^, and these values are comparable to our MEANS-ST1.0 estimates of 3.8 Tg and 0.4 Tg, respectively. Based on the coupled human-nature system (CHANS) model, Gu *et al*. calculated the total nitrogen budget for China in 2017; the total nitrogen discharge to the surface water system was 0.8 Tg, which is slightly greater than our estimate because the accounting of CHANS covers all natural and anthropogenic inputs. Simultaneously, the components of CHANS encompass crop farming, livestock farming, residential wastewater and industrial inputs to the surface water system, and these are highly similar to the components used in our estimates^66^. Our estimates are lower than those of the IMAGE and MARINA models, primarily because these methods overestimate nutrient discharge factors in China by 3–6 times compared to the 2017 Second National Pollutant Source Census data^27,67,68^. At a regional scale, Liu *et al*. developed a high-resolution nutrient discharge inventory (CEIN) for the Yangtze River Basin and evaluated point source nutrient pollution, such as urban wastewater treatment, industrial and centralized livestock farming discharge, for the year 2017. The TN and TP industrial discharge estimates of 75.0 Gg and 3.0 Gg^69^ in their study are very similar to our MEANS-ST1.0 industrial discharge estimates of 74.9 Gg and 3.7 Gg, respectively. Zhang *et al*. calculated the anthropogenic nitrogen discharge from human activities in the Yangtze River Delta for the year 2020, reporting crop farming, livestock farming and residential discharge values of 0.13 Tg, 0.30 Tg and 0.25 Tg^70^, respectively, which are commensurate with our MEANS-ST1.0 estimates of 0.14 Tg, 0.15 Tg and 0.21 Tg, respectively.

In addition, the spatiotemporal distribution of the MEANS-ST1.0 data is similar to that in other studies. Spatially, we generated high-resolution (1 km × 1 km) monthly maps of the anthropogenic discharge of total nitrogen (TN) and total phosphorus (TP) from different sources in China in 1980, 2000, and 2020, revealing that the pollutant discharge intensities in Shandong, Henan, Guangdong, Guangxi and Sichuan provinces are currently among the highest. Gu *et al*. also produced a high-resolution map of Nr inputs in 2017, identifying hotspots in the North China Plain region, the middle and lower reaches of the Yangtze River, and the Sichuan Basin^66^. Chen *et al*. developed nutrient input maps at different spatial scales (subbasin, grid, county and polygon) for China in 2012; the authors indicated that the southern and eastern regions of China, which are characterized by intensive agriculture and high urbanization rates, produce higher nitrogen and phosphorus inputs to rivers than other regions, with the Shandong, Hebei, Henan, and Guangxi and Guangdong coastal areas contributing more to river nutrient inputs than other regions^71^. In summary, the spatial distributions reported in these studies are similar to ours. Temporally, Ma *et al*. found that anthropogenic pollutant discharge has been continuously decreasing since 2003, mainly due to significant reductions in discharge from the urban and rural residential sectors. However, growing discharge from the livestock farming sector threatens these gains^72^, which is consistent with our findings (Fig. 4). Moreover, anthropogenic nutrient discharge is influenced by seasonal factors, such as climate and meteorology, daily life patterns, and agricultural practices; these factors exhibit certain dynamic patterns among different seasons and months, which is consistent with the findings of Meals *et al*.^30,73^. Overall, the MEANS-ST1.0 database provides high-quality and open-access information on anthropogenic nutrient discharge in China.

### Supplementary information

Supplementary Information

## Data Availability

All calculations were done with the help of ESRI ArcGIS 10.7, and no computer code is used to generate the data in the manuscript.
