# Supplementary material for: High resolution spatiotemporal modeling of long term anthropogenic nutrient discharge in China
Source: Sci Data. 2024 Mar 9;11:283. doi: 10.1038/s41597-024-03102-9 (PMC10925032; doi:10.1038/s41597-024-03102-9)
Supplement: Supplementary file 1 — Supplementary Information [file 41597_2024_3102_MOESM1_ESM.docx]

Supplementary Information for

**High resolution spatiotemporal modeling of long term anthropogenic nutrient discharge in China**

Haoran Zhang^a^, Huihang Sun^a^, Ruikun Zhao^a^, Yu Tian^a, *^, and Yiming Meng^a^

^a^ State Key Lab of Urban Water Resource and Environment, School of Environment, Harbin Institute of Technology, Harbin 150090, China

*Corresponding author. School of Environment, P.O.Box 2603, 73 Huanghe Road, Nangang District, Harbin, Heilongjiang Province 150090, China

E-mail address: hit_tianyu@163.com; Tel: +0086-451-86283077

Number of pages: 19

Number of figures: 4

Number of tables: 10

Contents

[Figures 3](#_Toc159926282)

[Figure S1 Cold-hot spot analysis of total anthropogenic nutrient discharge in China from 1980 to 2020. 3](#_Toc159926283)

[Figure S2 Temporal changes in total anthropogenic nutrient discharge and their components in China from 1980 to 2020. 4](#_Toc159926284)

[Figure S3 Relationship between industrial TN discharge and industrial GDP. 5](#_Toc159926285)

[Figure S4 Relationship between industrial TP discharge and industrial GDP. 6](#_Toc159926286)

[Tables 7](#_Toc159926287)

[Table S1 Changes of cold-hot spots of anthropogenic nutrient discharge between 1980 and 2020. 7](#_Toc159926288)

[Table S2 Parameters characteristics and data sources of MEANS-ST1.0. 8](#_Toc159926289)

[Table S3 Pollutant concentrations in direct discharge water and pollutant concentrations in discharge water from urban WWTPs by province, mg·L^-1^. 10](#_Toc159926290)

[Table S4 Proportion of flush toilet used in different provinces of China in 1980, 2000 and 2020, %. 11](#_Toc159926291)

[Table S5 Pollutant generation coefficient of rural residents, mg·L^-1^. 12](#_Toc159926292)

[Table S6 Pollutant removal efficiency of rural wastewater treatment, %. 13](#_Toc159926293)

[Table S7 Loss coefficients of nitrogen and phosphorus pollutants from farmland in each province, kg ha^-1^. 14](#_Toc159926294)

[Table S8 Summary of pastoral description and pastoral farming ratio, %. 15](#_Toc159926295)

[Table S9 Matrix used for the data quality assessment. 16](#_Toc159926296)

[Table S10 Comparison of MEANS-ST1.0 results with those of other studies. 17](#_Toc159926297)

[References 18](#_Toc159926298)

# Figures

## Figure S1 Cold-hot spot analysis of total anthropogenic nutrient discharge in China from 1980 to 2020.


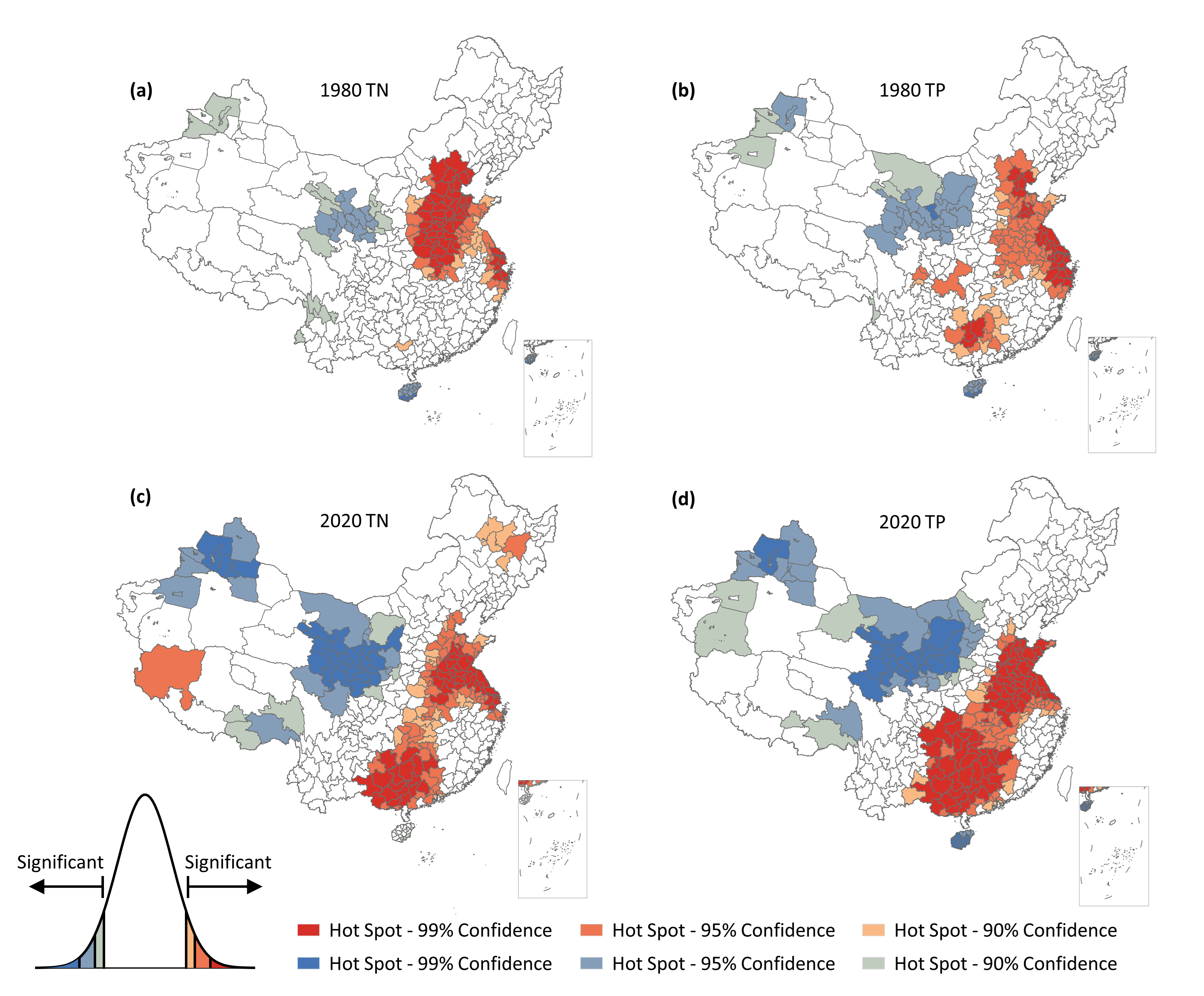


Figure S1 Cold-hot spot analysis of total anthropogenic nutrient discharge in China from 1980 to 2020. (a) Anthropogenic TN discharge in 1980; (b) Anthropogenic TP discharge in 1980; (c) Anthropogenic TN discharge in 2020; (d) Anthropogenic TP discharge in 2020. The study employs a total of 367 cities throughout the country as statistical units to conduct cold-hot spot analysis. There are no data for Hong Kong, Macao, and Taiwan.

## Figure S2 Temporal changes in total anthropogenic nutrient discharge and their components in China from 1980 to 2020.


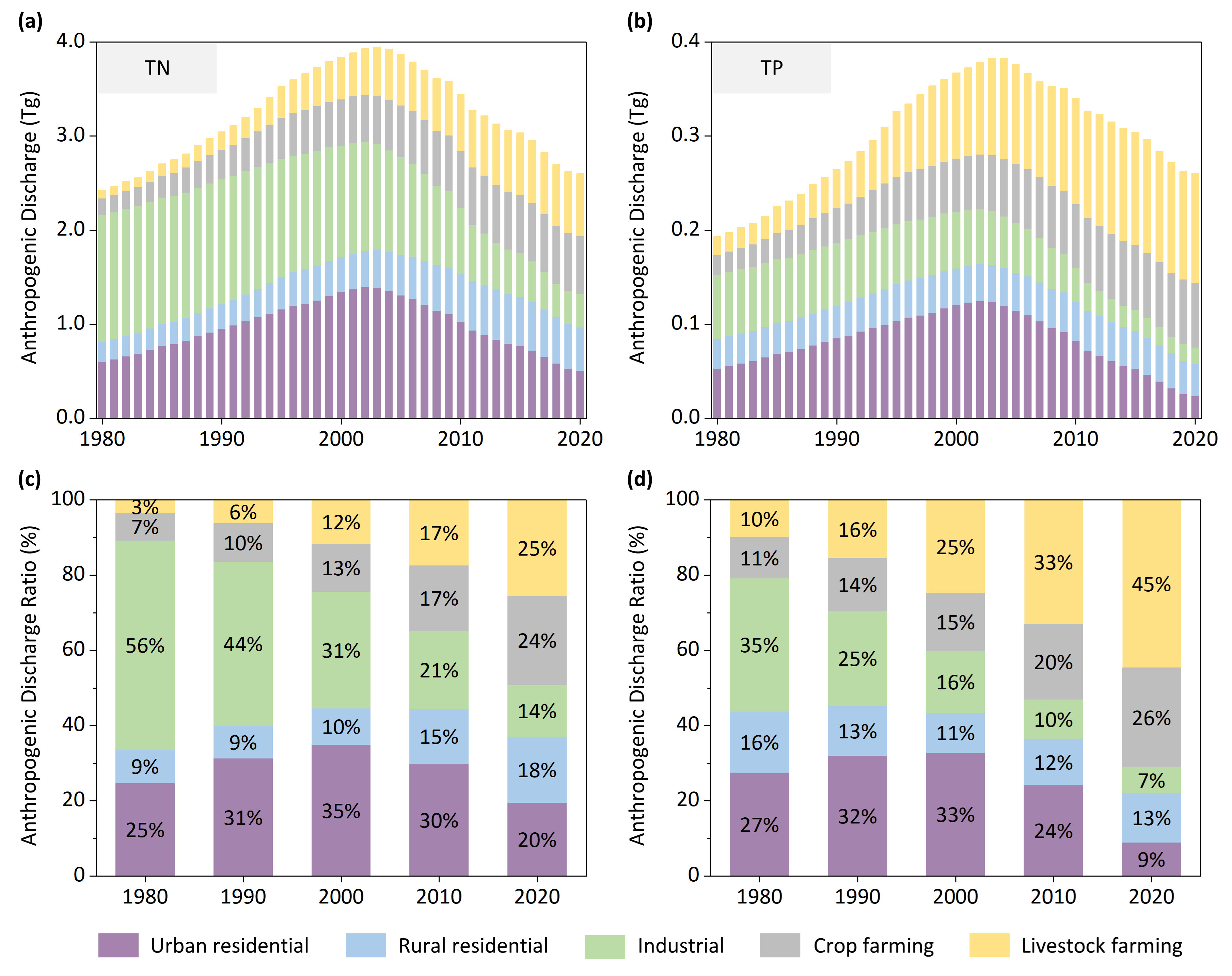


Figure S2 Temporal changes in total anthropogenic nutrient discharge and their components in China from 1980 to 2020. (a) Anthropogenic TN discharge; (b) Anthropogenic TP discharge; (c) Components of Anthropogenic TN discharge; (d) Components of Anthropogenic TP discharge. There are no data for Hong Kong, Macao, and Taiwan.

## Figure S3 Relationship between industrial TN discharge and industrial GDP.


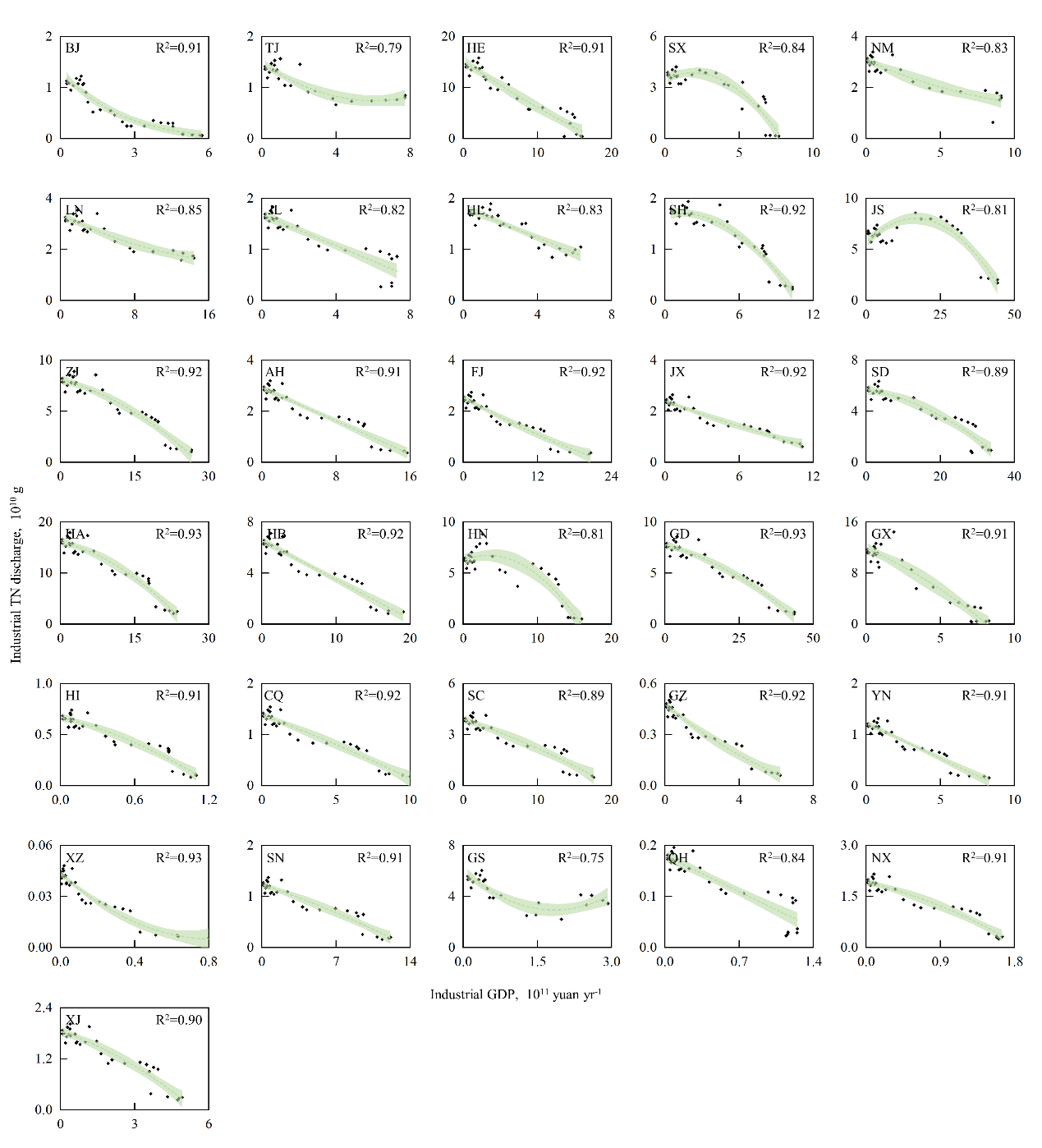


## Figure S4 Relationship between industrial TP discharge and industrial GDP.


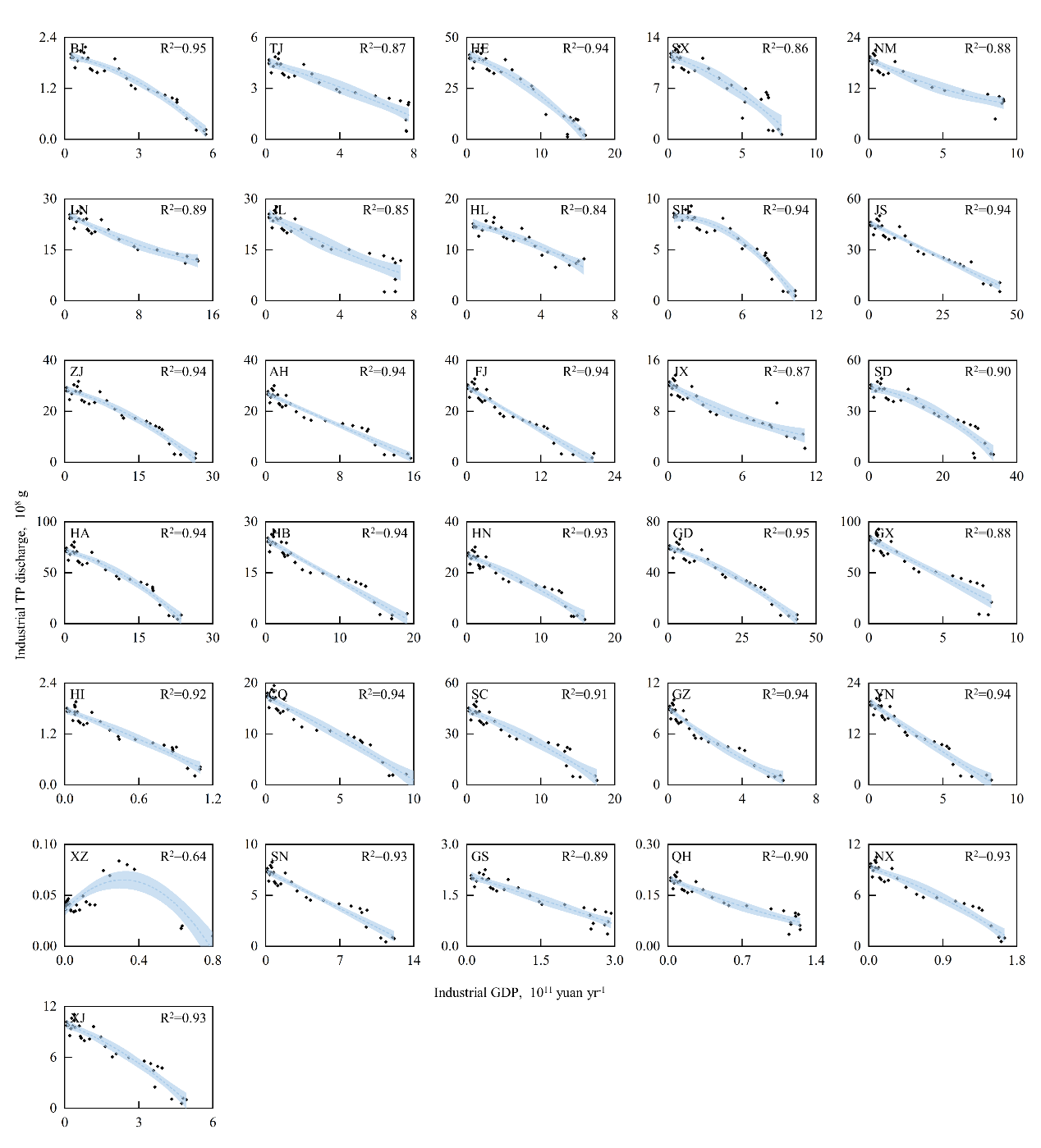


# Tables

## Table S1 Changes of cold-hot spots of anthropogenic nutrient discharge between 1980 and 2020.

| Pollutant | Category | Number of cities |
| --- | --- | --- |
| TN | Persistent hotspot | 64 |
|  | Persistent coldspot | 25 |
|  | Newly emerged hotspots | 62 |
|  | Newly emerged coldspot | 36 |
| TP | Persistent hotspot | 83 |
|  | Persistent coldspot | 55 |
|  | Newly emerged hotspots | 48 |
|  | Newly emerged coldspot | 27 |

## Table S2 Parameters characteristics and data sources of MEANS-ST1.0.

| **Department** | **Meaning** | **Symbol** | **Unit** | **Data change characteristics** | **Data Sources** |
| --- | --- | --- | --- | --- | --- |
| Urban residential | Urban population | $\text{U}_{\text{pop}}$ | person | Spatial and temporal | [1] China Statistical Yearbook^1^  [2] Provincial Statistical Yearbooks^2^ |
|  | Per capita urban residential water consumption | $\text{UM}_{\text{water}}$ | L/day/person | Spatial | [1] China Statistical Yearbook^1^  [2] Provincial Statistical Yearbooks^2^ |
|  | Urban wastewater treatment rate | $\text{UR}_{\text{treat}}$ | % | Spatial and temporal | [1] China Urban-Rural Construction Statistical Yearbook^3^ |
|  | Urban wastewater reuse rate | $\text{UR}_{\text{reuse}}$ | % | Spatial and temporal | [1] China Statistical Yearbook on Environment^4^ |
|  | Pollutant discharge concentration of direct discharge wastewater | $\text{UC}_{\text{direct}}$ | mg/L | Spatial | [1] Second National Pollutant Source Census^5^ |
|  | Pollutant discharge concentration of WWTPs | $\text{UC}_{\text{treat}}$ | mg/L | Spatial and temporal | [1] Second National Pollutant Source Census^5^  [2] Report on the Development of Urban Sewage Treatment and Recycling in China^6^ |
| Rural residential | Rural population | $\text{R}_{\text{pop}}$ | person | Spatial and temporal | [1] China Statistical Yearbook^1^  [2] Provincial Statistical Yearbooks^2^ |
|  | Flush toilets ratio | $\text{RR}_{\text{flu}}$ | % | Spatial and temporal | [1] China Statistical Yearbook on Environment^4^ |
|  | Pollutant generation coefficient of rural residents using dry toilets | $\text{RM}_{\text{dry}}$ | g/day/person | Spatial | [1] Second National Pollutant Source Census^5^ |
|  | Pollutant generation coefficient of rural residents using flush toilets | $\text{RM}_{\text{flu}}$ | g/day/person | Spatial | [1] Second National Pollutant Source Census^5^ |
|  | Rural wastewater treatment rate | $\text{RM}_{\text{treat}}$ | % | Spatial and temporal | [1] China Urban-Rural Construction Statistical Yearbook^3^ |
|  | Pollutant removal rate of rural wastewater treatment | $\text{RM}_{\text{removal}}$ | % | Spatial | [1] Second National Pollutant Source Census^5^ |
| Industrial | Industrial discharge | $\text{ID}$ | Gg/yr | Spatial and temporal | [1] China Statistical Yearbook on Environment^4^ |
|  | Industrial GDP | GDP | yuan | Spatial and temporal | [1] China Statistical Yearbook^1^  [2] Provincial Statistical Yearbooks^2^ |
| Farming | Total sown area | $\text{F}_{\text{area}}$ | ha | Spatial and temporal | [1] China Statistical Yearbook^1^  [2] Provincial Statistical Yearbooks^2^ |
|  | Fertilizer application | $\text{F}_{\text{fertilizer}}$ | t | Spatial and temporal | [1] China Statistical Yearbook^1^  [2] Provincial Statistical Yearbooks^2^ |
|  | Pollutant loss coefficient of farmland | $\text{FM}$ | kg/ha | Spatial | [1] Second National Pollutant Source Census^5^ |
| Livestock | Number of fattened stock | $\text{L}_{\text{num}}$ | head | Spatial and temporal | [1] China Statistical Yearbook^1^  [2] Provincial Statistical Yearbooks^2^ |
|  | Centralized farming rate | $\text{LR}_{\text{centralized}}$ | % | Temporal | [1] China Animal Husbandry and Veterinary Yearbook^7^ |
|  | Pollutant discharge coefficient for centralized farming | $\text{LM}_{\text{centralized}}$ | kg/head | Spatial | [1] Second National Pollutant Source Census^5^ |
|  | Pollutant discharge coefficient for free-range farming | $\text{LM}_{\text{free}}$ | kg/head | Spatial | [1] Second National Pollutant Source Census^5^ |
|  | Pastoral farming ratio | $\text{LR}_{\text{pastoral}}$ | % | Spatial | [1] China Animal Husbandry and Veterinary Yearbook^7^ |

## Table S3 Pollutant concentrations in direct discharge water and pollutant concentrations in discharge water from urban WWTPs by province, mg·L^-1^.

| Province  (Abbreviation) | Pollutant concentrations in direct discharge water | | Pollutant concentrations in effluent from urban WWTPs | |
| --- | --- | --- | --- | --- |
|  | TN | TP | TN | TP |
| Beijing, BJ | 73.80 | 5.80 | 9.10 | 0.60 |
| Tianjin, TJ | 73.80 | 5.80 | 9.70 | 0.50 |
| Hebei, HE | 73.80 | 5.80 | 8.10 | 0.40 |
| Shanxi, SX | 73.80 | 5.80 | 9.50 | 0.50 |
| Inner Mongolia, NM | 62.10 | 4.80 | 14.90 | 0.60 |
| Liaoning, LN | 48.70 | 4.40 | 7.60 | 0.50 |
| Jilin, JL | 48.70 | 4.40 | 9.40 | 0.50 |
| Heilongjiang, HL | 48.70 | 4.40 | 11.30 | 0.60 |
| Shanghai, SH | 44.80 | 4.30 | 8.60 | 0.70 |
| Jiangsu, JS | 44.80 | 4.30 | 8.10 | 0.50 |
| Zhejiang, ZJ | 44.80 | 4.30 | 8.40 | 0.50 |
| Anhui, AH | 44.80 | 4.30 | 7.60 | 0.40 |
| Fujian, FJ | 44.80 | 4.30 | 7.80 | 0.40 |
| Jiangxi, JX | 44.80 | 4.30 | 5.80 | 0.30 |
| Shandong, SD | 73.80 | 5.80 | 11.40 | 0.70 |
| Henan, HA | 73.80 | 5.80 | 7.90 | 0.40 |
| Hubei, HB | 39.40 | 4.10 | 7.10 | 0.40 |
| Hunan, HN | 39.40 | 4.10 | 6.80 | 0.30 |
| Guangdong, GD | 39.40 | 4.10 | 6.00 | 0.40 |
| Guangxi, GX | 39.40 | 4.10 | 7.30 | 0.40 |
| Hainan, HI | 39.40 | 4.10 | 6.10 | 0.40 |
| Chongqing, CQ | 49.80 | 4.30 | 9.40 | 0.70 |
| Sichuan, SC | 49.80 | 4.30 | 10.40 | 0.60 |
| Guizhou, GZ | 49.80 | 4.30 | 8.20 | 0.50 |
| Yunnan, YN | 49.80 | 4.30 | 9.00 | 0.60 |
| Tibet, XZ | 49.80 | 4.30 | 4.80 | 0.40 |
| Shaanxi, SN | 71.20 | 5.10 | 13.10 | 0.50 |
| Gansu, GS | 71.20 | 5.10 | 20.20 | 0.60 |
| Qinghai, QH | 71.20 | 5.10 | 12.00 | 0.40 |
| Ningxia, NX | 71.20 | 5.10 | 16.80 | 0.70 |
| Xinjiang, XJ | 71.20 | 5.10 | 15.00 | 0.40 |

## Table S4 Proportion of flush toilet used in different provinces of China in 1980, 2000 and 2020, %.

| Province | 1980 | 2000 | 2020 |
| --- | --- | --- | --- |
| BJ | 2.50 | 23.40 | 99.40 |
| TJ | 0.00 | 0.00 | 93.20 |
| HE | 0.30 | 6.70 | 55.40 |
| SX | 0.40 | 4.70 | 44.60 |
| NM | 0.00 | 0.20 | 32.20 |
| LN | 0.30 | 6.10 | 40.30 |
| JL | 0.00 | 0.30 | 20.10 |
| HL | 0.50 | 4.40 | 16.30 |
| SH | 83.40 | 92.60 | 99.40 |
| JS | 0.00 | 10.80 | 95.40 |
| ZJ | 0.00 | 36.20 | 98.80 |
| AH | 1.40 | 10.20 | 57.00 |
| FJ | 0.00 | 44.10 | 100.00 |
| JX | 4.90 | 21.90 | 85.80 |
| SD | 1.60 | 16.70 | 75.50 |
| HA | 25.60 | 41.70 | 61.40 |
| HB | 0.00 | 23.70 | 63.40 |
| HN | 0.00 | 21.20 | 45.90 |
| GD | 21.50 | 53.30 | 95.80 |
| GX | 8.10 | 30.80 | 88.40 |
| HI | 15.20 | 42.60 | 87.60 |
| CQ | 4.00 | 18.50 | 80.20 |
| SC | 4.80 | 20.40 | 75.70 |
| GZ | 0.40 | 4.40 | 60.70 |
| YN | 1.50 | 11.20 | 61.60 |
| XZ | 0.00 | 0.00 | 0.00 |
| SN | 4.10 | 14.80 | 53.10 |
| GS | 0.50 | 4.90 | 44.00 |
| QH | 0.20 | 2.20 | 16.00 |
| NX | 0.00 | 0.00 | 58.00 |
| XJ | 0.00 | 3.30 | 77.80 |

## Table S5 Pollutant generation coefficient of rural residents, mg·L^-1^.

| Province | Dry toilet | | Flush toilet | |
| --- | --- | --- | --- | --- |
|  | TN | TP | TN | TP |
| BJ | 0.46 | 0.12 | 4.46 | 0.23 |
| TJ | 0.46 | 0.12 | 4.46 | 0.23 |
| HE | 0.30 | 0.04 | 2.66 | 0.11 |
| SX | 0.28 | 0.03 | 2.52 | 0.10 |
| NM | 0.48 | 0.06 | 3.68 | 0.24 |
| LN | 0.64 | 0.09 | 3.51 | 0.18 |
| JL | 0.61 | 0.08 | 3.26 | 0.17 |
| HL | 0.65 | 0.09 | 3.52 | 0.18 |
| SH | 0.75 | 0.15 | 5.07 | 0.34 |
| JS | 0.72 | 0.14 | 4.10 | 0.31 |
| ZJ | 0.74 | 0.15 | 4.74 | 0.33 |
| AH | 0.71 | 0.14 | 3.71 | 0.30 |
| FJ | 0.71 | 0.14 | 3.76 | 0.30 |
| JX | 0.70 | 0.14 | 3.67 | 0.30 |
| SD | 0.36 | 0.07 | 3.37 | 0.16 |
| HA | 0.31 | 0.04 | 2.78 | 0.12 |
| HB | 0.75 | 0.15 | 6.00 | 0.46 |
| HN | 0.72 | 0.13 | 5.78 | 0.44 |
| GD | 0.75 | 0.15 | 5.97 | 0.46 |
| GX | 0.73 | 0.14 | 5.88 | 0.45 |
| HI | 0.77 | 0.16 | 6.23 | 0.48 |
| CQ | 0.47 | 0.08 | 4.43 | 0.31 |
| SC | 0.47 | 0.08 | 4.71 | 0.33 |
| GZ | 0.46 | 0.08 | 3.89 | 0.29 |
| YN | 0.46 | 0.08 | 3.86 | 0.28 |
| XZ | 0.46 | 0.08 | 3.77 | 0.28 |
| SN | 0.31 | 0.03 | 2.47 | 0.19 |
| GS | 0.32 | 0.03 | 2.72 | 0.21 |
| QH | 0.31 | 0.03 | 2.53 | 0.20 |
| NX | 0.34 | 0.04 | 3.00 | 0.23 |
| XJ | 0.36 | 0.04 | 3.34 | 0.26 |

## Table S6 Pollutant removal efficiency of rural wastewater treatment, %.

| Province | TN | TP |
| --- | --- | --- |
| BJ | 47.00 | 46.00 |
| TJ | 47.00 | 46.00 |
| HE | 47.00 | 46.00 |
| SX | 47.00 | 46.00 |
| NM | 47.00 | 46.00 |
| LN | 46.00 | 46.00 |
| JL | 46.00 | 46.00 |
| HL | 46.00 | 46.00 |
| SH | 46.00 | 48.00 |
| JS | 46.00 | 48.00 |
| ZJ | 46.00 | 48.00 |
| AH | 46.00 | 48.00 |
| FJ | 46.00 | 48.00 |
| JX | 46.00 | 48.00 |
| SD | 45.00 | 45.00 |
| HA | 47.00 | 46.00 |
| HB | 46.00 | 48.00 |
| HN | 46.00 | 48.00 |
| GD | 46.00 | 48.00 |
| GX | 46.00 | 48.00 |
| HI | 46.00 | 48.00 |
| CQ | 46.00 | 47.00 |
| SC | 46.00 | 47.00 |
| GZ | 46.00 | 47.00 |
| YN | 46.00 | 47.00 |
| XZ | 46.00 | 47.00 |
| SN | 43.00 | 46.00 |
| GS | 43.00 | 46.00 |
| QH | 43.00 | 46.00 |
| NX | 43.00 | 46.00 |
| XJ | 43.00 | 46.00 |

## Table S7 Loss coefficients of nitrogen and phosphorus pollutants from farmland in each province, kg ha^-1^.

| Province | TN | TP |
| --- | --- | --- |
| BJ | 0.83 | 0.06 |
| TJ | 1.09 | 0.09 |
| HE | 0.78 | 0.07 |
| SX | 0.87 | 0.06 |
| NM | 0.12 | 0.02 |
| LN | 0.74 | 0.06 |
| JL | 0.53 | 0.02 |
| HL | 1.03 | 0.10 |
| SH | 6.06 | 0.77 |
| JS | 6.48 | 0.70 |
| ZJ | 15.02 | 2.45 |
| AH | 5.42 | 0.57 |
| FJ | 11.19 | 1.45 |
| JX | 6.68 | 0.86 |
| SD | 0.82 | 0.02 |
| HA | 2.98 | 0.23 |
| HB | 6.68 | 0.79 |
| HN | 6.30 | 0.58 |
| GD | 11.55 | 1.62 |
| GX | 11.43 | 1.43 |
| HI | 11.27 | 1.51 |
| CQ | 3.60 | 0.40 |
| SC | 3.40 | 0.37 |
| GZ | 3.56 | 0.57 |
| YN | 6.39 | 0.51 |
| XZ | 0.18 | 0.02 |
| SN | 1.49 | 0.18 |
| GS | 0.40 | 0.03 |
| QH | 0.23 | 0.02 |
| NX | 0.19 | 0.02 |
| XJ | 0.22 | 0.01 |

## Table S8 Summary of pastoral description and pastoral farming ratio, %.

| Province | Beef cattle | Dairy cow | Sheep |
| --- | --- | --- | --- |
| HE | 36.61 | 56.43 | 9.18 |
| SX | 3.11 | 3.62 | 6.90 |
| NM | 76.77 | 100.00 | 57.33 |
| LN | 43.98 | 35.04 | 100.00 |
| JL | 15.40 | 15.40 | 100.00 |
| HL | 33.62 | 32.66 | 50.87 |
| SC | 51.08 | 65.21 | 30.08 |
| YN | 1.06 | 2.18 | 0.75 |
| XZ | 82.99 | 73.02 | 84.59 |
| GS | 34.91 | 37.95 | 49.52 |
| QH | 75.17 | 87.91 | 78.36 |
| NX | 35.34 | 42.04 | 53.77 |
| XJ | 46.02 | 48.44 | 37.19 |

## Table S9 Matrix used for the data quality assessment.

| **Score** | **Data acquisition** | **Data independence** | **Data representation** | **Data age** | **Geographical correlation** | **Technological correlation** |
| --- | --- | --- | --- | --- | --- | --- |
| 1 | Measured data | Verified data, information from public or other independent source | Representative data from sufficient samples of sites over an adequate period | Less than 3 years of difference to years of study | Data from study area | Data from enterprises, processes, and materials under study |
| 2 | Calculated data based on measurements | Verified information from enterprises with interest in the study | Representative data from smaller number of sites but for adequate periods | Less than 5 years of difference to years of study | Average data from larger area in which the study area is included | Data from processes and materials under study but from different enterprises |
| 3 | Calculated data partly based on assumptions | Independent source, but based on non-verified information from industry | Representative data from adequate sites, but over shorter periods | Less than 10 years of difference to years of study | Data from area with similar production conditions | Data from processes and materials under study but from different technology |
| 4 | Qualified estimate by industrial expert | Non-verified information from industry | Data from adequate number of sites but shorter periods | Less than 20 years of differences to year of study | Data from area with slightly similar production conditions | Data on related processes or materials but same technology |
| 5 | Non-qualified estimate | Non-verified information from the enterprise interested in this study | Representativeness unknown or income-plete data from smaller number of sites and/or from shorter periods, age unknown or more than 20 years of difference | Age unknown or more than 20 years of difference | Data from unknown area or with very different production conditions | Data on related processes or materials but different technology |

## Table S10 Comparison of MEANS-ST1.0 results with those of other studies.

| Pollutant | Area | Discharge/Tg | Year | Study |
| --- | --- | --- | --- | --- |
| TN | China | 2.42-3.95 | 1980-2020 | MEANS-ST1.0 |
|  |  | 4.65 | 2007 | First National  Pollutant Source Census^8^ |
|  |  | 3.04 | 2017 | Second National Pollutant Source Census^5^ |
|  |  | 8.7 | 2017 | CHANS^1^ |
|  |  | 26.5 | 2017 | Yu et al.^9^ |
|  |  | 27.8 | 2012 | Chen et al.^10^ |
|  | North America | 2.53 | 2000 | Global NEWS^11^ |
|  | Central and South America | 1.89 |  |  |
|  | Europe | 2.96 |  |  |
|  | Africa | 2.79 |  |  |
|  | North Asia | 0.73 |  |  |
|  | South Asia | 15.0 |  |  |
|  | Eastern Asia China and Mongolia | 5.36 |  |  |
|  | Oceania | 0.15 |  |  |
|  | Global | 33.4 | 2000 | Morée et al.^12^ |
|  |  | 21.8 | 1980 |  |
| TP | China | 0.19-0.38 | 1980-2020 | MEANS-ST1.0 |
|  |  | 0.41 | 2007 | First National  Pollutant Source Census^8^ |
|  |  | 0.32 | 2017 | Second National Pollutant Source Census^5^ |
|  |  | 2.92 | 2012 | Chen et al.^10^ |
|  | North America | 0.42 | 2000 | Global NEWS^11^ |
|  | Central and South America | 0.34 |  |  |
|  | Europe | 0.52 |  |  |
|  | Africa | 0.48 |  |  |
|  | North Asia | 0.13 |  |  |
|  | South Asia | 2.63 |  |  |
|  | Eastern Asia China and Mongolia | 0.91 |  |  |
|  | Oceania | 0.03 |  |  |
|  | Global | 4.3 | 2000 | Morée et al.^12^ |
|  |  | 3.2 | 1980 |  |

# References

1 National Bureau of Statistics of the People’s Republic of China. *China Statistical Yearbook* [in Chinese]. (China Statistics Press, 2021).

2 National Bureau of Statistics of the People’s Republic of China. *Statistical Yearbooks of provinces* [in Chinese]. (China Statistics Press, 2021).

3 Ministry of Housing and Urban-Rural Development of the People's Republic of China. *China Urban-Rural Construction Statistical Yearbook* [in Chinese]. (Beijing China Planning Publishing House, 2021).

4 National Bureau of Statistics of the People’s Republic of China. *China Statistical Yearbook on Environment* [in Chinese]. (China Statistics Press, 2021).

5 National Bureau of Statistics of the People’s Republic of China. *The Second National Pollutant Source Census in 2017* [in Chinese]. (China Statistics Press, 2018).

3 China Civil Engineering Society & Chinese Society for Environmental Sciences. *Report on the Development of Urban Sewage Treatment and Recycling in China* [in Chinese]. (China Construction Industry Press, 2020).

7 National Bureau of Statistics of the People’s Republic of China. *China Animal Husbandry and Veterinary Yearbook* [in Chinese]. (China Agriculture Press, 2021).

8 National Bureau of Statistics of the People’s Republic of China. The First National Pollutant Source Census in 2007 [in Chinese]. (China Statistics Press, 2008).

9 Yu, C. *et al.* Managing nitrogen to restore water quality in China. Nature 567, 516-520, doi:10.1038/s41586-019-1001-1 (2019).

10 Chen, X. *et al.* Multi-scale Modeling of Nutrient Pollution in the Rivers of China. Environ Sci Technol 53, 9614-9625, doi:10.1021/acs.est.8b07352 (2019).

11 Van Drecht, G., Bouwman, A. F., Harrison, J. & Knoop, J. M. Global nitrogen and phosphate in urban wastewater for the period 1970 to 2050. Global Biogeochemical Cycles 23, n/a-n/a, doi:10.1029/2009gb003458 (2009).

12 Morée, A. L., Beusen, A. H. W., Bouwman, A. F. & Willems, W. J. Exploring global nitrogen and phosphorus flows in urban wastes during the twentieth century. Global Biogeochemical Cycles 27, 836-846, doi:10.1002/gbc.20072 (2013).
